# Phenotypic characterization of an *Atp13a2* knockout rat model of Parkinson’s disease

**DOI:** 10.1038/s41531-025-01171-0

**Published:** 2025-11-18

**Authors:** Rémi Kinet, Joanna Sikora, Marie-Laure Arotcarena, Melina Decourt, Eric Balado, Evelyne Doudnikoff, Sylvain Bohic, Marta Vesnaver, Anna Lovisotto, Marie-Laure Thiolat, Nathalie Dutheil, Claire Mazzocco, Karim Harhouri, Rémy Steinschneider, Severine Menoret, Laurent Tesson, Ignacio Anegon, Michele Morari, Miquel Vila, François Georges, Erwan Bezard, Pierre-Olivier Fernagut, Benjamin Dehay

**Affiliations:** 1 Univ. de Bordeaux, CNRS, IMN, UMR 5293, Bordeaux, France; 2 Univ. De Poitiers, INSERM, LNEC, U-1084, Poitiers, France; 3 Synchrotron Radiation for Biomedicine (STROBE), Univ. Grenoble Alpes, Inserm, UA7, Grenoble, France; 4 Institute of Applied Physiology, Ulm University, Ulm, Germany; 5 Neuron Experts SAS, Marseille, France; 6 Nantes Université, Inserm, Centre de Recherche en Transplantation et Immunologie, UMR 1064, CNRS, SFR Santé, Inserm UMS 016 CNRS UMS 3556, Nantes, France; 7 Nantes Université, CHU Nantes, INSERM, Center for Research in Transplantation and Translational Immunology, UMR 1064, Nantes, France; 8 Nantes Université, Inserm, Centre de Recherche en Transplantation et Immunologie, UMR 1064, Nantes, France; 9 Department of Pharmaceutical and Pharmacological Sciences, Section of Pharmacology, University of Padova, Padova, Italy; 10 Center for Networked Biomedical Research on Neurodegenerative Diseases (CIBERNED), Instituto Carlos III, Madrid, Spain; 11 Neurodegenerative Diseases Research Group, Vall d’Hebron Research Institute (VHIR), Barcelona, Spain; 12 Department of Biochemistry and Molecular Biology, Autonomous University of Barcelona (UAB), Barcelona, Spain; 13 Catalan Institution for Research and Advanced Studies (ICREA), Barcelona, Spain

**Keywords:** Cell biology, Diseases, Molecular biology, Neurology, Neuroscience

## Abstract

Mutations in the *ATP13A2* gene were identified as the cause of Kufor-Rakeb syndrome (KRS), a juvenile-onset form of Parkinson’s disease (PD). Developing relevant and predictive models for the rare PD forms is necessary to understand the pathological mechanisms and validate therapeutic strategies. Herein, we aimed to comprehensively characterize the first transgenic *Atp13a2* knockout rat model. Behavioral assessment demonstrated specific developmental deficits in animals with deletion of *Atp13a2*. Further analysis revealed that *Atp13a2* knockout rats displayed age-dependent fine motor skills deficits and impaired locomotor habituation similar to those observed in PD patients at the early stage of motor symptoms. In contrast, no change in the nigrostriatal integrity was observed. An extended investigation on heavy metals homeostasis, autophagy-related markers, and lipofuscin accumulation showed significant changes reminiscent of KRS. Finally, we tested whether inducing pathology by viral-mediated overexpression of human α-synuclein or human tyrosinase exacerbated the onset or extent of pathological changes. This *Atp13a2* KO rat model could help better understand autophagy in PD pathogenesis and open new therapeutic validation opportunities.

## Introduction

Parkinson’s disease (PD) is the most common motor neurodegenerative disorder, characterized by the loss of dopaminergic innervation in the nigrostriatal pathway and the presence of α-synuclein (α-syn)-positive intraneuronal inclusions called Lewy Bodies (LB)^1,2^. Even if most PD cases are sporadic, around 10% of patients are affected by a familial type associated with inherited genomic mutations^3^. The *ATP13A2* gene, encoding a transmembrane lysosomal P5B-type ATPase protein, has been identified as the cause of an autosomal recessive juvenile-onset form of PD called Kufor-Rakeb syndrome (KRS)^4^. This pathology is characterized by the emergence of symptoms, usually paraparesis, akinesia, rigidity, hyperreflexia, ocular disabilities, and dementia at the age of ~15^5,6^. Patients typically die around the age of 30, making ATP13A2 dysfunction a very aggressive pathological factor. Indeed, ATP13A2 is involved in transporting polyamines, which are organic polycations^7–9^. While ATP13A2 has been linked to sensitivity to specific divalent metal ions such as Mn^2+^ and Zn^2+^, these are not considered direct transport substrates^10^. Furthermore, *ATP13A2* mutations cause neurodegeneration associated with iron accumulation^11^. This protein is also implicated in autophagy by regulating lysosomal pH and catabolism^12^. PD-linked *ATP13A2* mutations increase lumen pH in the lysosome, thereby reducing lysosomal enzymatic activity, substrate degradation, and autophagy action^12^. Moreover, previous studies suggested a protective action of ATP13A2 against divalent heavy metal cations and α-syn accumulation^10,13^. Taken together, these features make ATP13A2 an important player in neuronal homeostasis, as it is also linked to amyotrophic lateral sclerosis^14^. The discovery of over 30 different pathogenic mutations in the *ATP13A2* gene^15^ makes it an attractive target for in vivo genetic modeling. Although less used than the mouse for genetic modeling, the rat remains a valuable model for neurodegenerative diseases such as PD, notably for its PD-related behavioral repertoire in other modeling modalities, and new genome editing technologies have now been applied to rat models^16^.

Several genetic and etiological rat models have been characterized, such as *PINK1*^17^, *DJ-1*, *Parkin*^18,19^, *LRRK2* KO rats^20,21^, but also LRRK2 G2019S transgenic rats^22,23^, or rats overexpressing wild-type or human E46K α-syn in BAC transgenic rats^24–28^ (for review, see ref. ^29^). Currently, in vivo, *Atp13a2* KO models are limited to different animal species^15^. *Atp13a2* KO mice exhibit subtle sensorimotor deficits, α-syn aggregation, and autophagy deficits^30^. Recently, a mouse with conditional deletion of *Atp13a2* in the SN^31^ and a non-human primate model where ATP13A2 expression was impaired by virally-delivered shATP13A2^32^ were reported to replicate a phenotype close to that observed in human KRS patients, with nigrostriatal neurodegeneration, neuroinflammation, and dysfunction of the autophagy-lysosomal pathway (ALP)^33^. There is, however, a lack of models of KRS and ATP13A2-related PD in rats. Here, we characterize the first CRISPR/Cas9-generated *Atp13a2* KO rat model to elucidate the mechanisms underlying ATP13A2-deficiency-associated pathology. We observed alterations in locomotor and fine motor skills, neuroinflammation, zinc dyshomeostasis, electrophysiological disturbances, and ALP dysfunctions in the SN without overt nigrostriatal degeneration.

## Results

### Generation of the Atp13A2 knockout rat

**Fig. 1:**
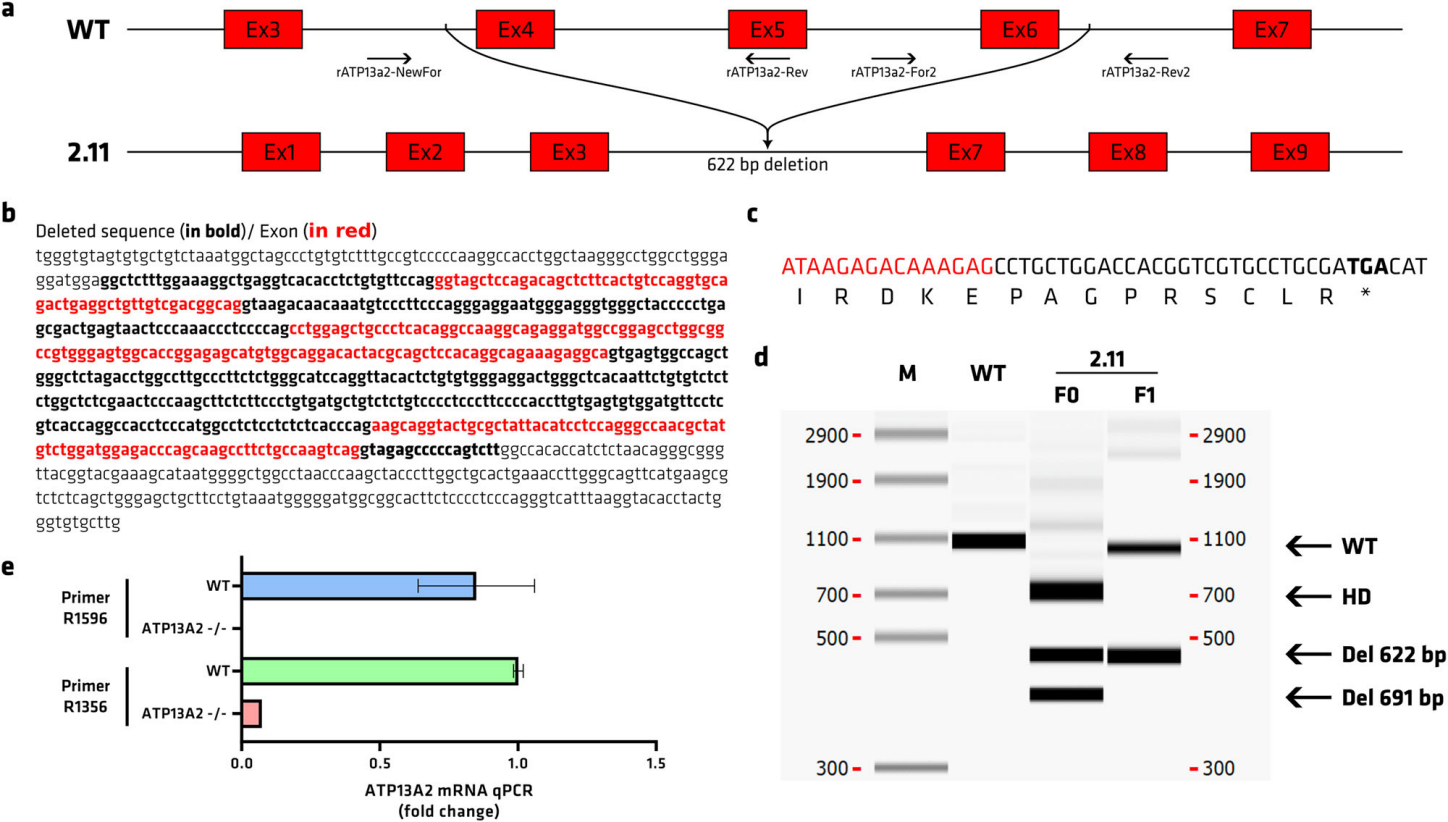
Generation of the *Atp13a2* knockout rat model. **a** Two sgRNA were designed and complexed with spCas9 protein to target sequences in intron 3 (sg937) and intron 6 (sg940). The expected deletion between the 2 sgRNA removes exons 4–6, leading to a frameshift and a premature termination codon in exon 7, which results in mRNA degradation of the whole transcript sequence via nonsense-mediated mRNA decay. Arrows indicate primers used for PCR analysis. **b** Sanger sequence of the targeted sequence and the sequence that was deleted in the 2.11 rat line. **c** mRNA analysis by RT-PCR showed a premature stop codon in exon 7 (in bold) immediately after exon 3 (in red). **d** Genotyping was done by PCR on tail genomic DNA, followed by an automated microfluidic chip capillary electrophoresis system (scale in base pairs). On PCR with primers NewFor and Rev2 (1062 bp the WT rat showed the expected band size, whereas the 2.11 F_0_ rat showed two bands with different deletions, one with a 691 bp deletion and the desired band of 622bp deletion corresponding to exons 4–6 deletion (the third band ~700 bp are heteroduplexes, HD). F_1_ heterozygous rat showed a WT band from the WT animal mated with the 2.11 F0 rat and a unique 622 bp deletion band. **e** Qualitative result of qPCR *Atp13a2* mRNA qPCR with primer 1596 or 1356 in WT or *Atp13a2*^−/−^.

The *Atp13a2* knockout (KO) rat model was generated with CRISPR/Cas9 technology to remove the *Atp13a2* gene sequence between exons 4 and 6 (Fig. 1a, b), leading to a frameshift and a premature termination codon in exon 7 (Fig. 1c). PCR on tail genomic DNA allowed the identification of WT and KO animals and confirmed the deletion of part of the *Atp13a2* gene (440 base pairs (bp) remaining sequences after the 622 bp deletion) in F_0_ founder and F_1_ heterozygous KO animals compared to the WT 1062 bp band (Fig. 1d). Due to this 622 bp deletion, a premature stop codon will appear and cause the mRNA to be degraded via Nonsense-Mediated mRNA Decay before it has a chance to produce the protein. Quantitative RT-PCR showed a significant reduction in mRNA levels or even an absence of transcripts with 2 sets of primers in homozygous animals of *Atp13a2* KO rats (Fig. 1e), indicating that the deletion mutations also destabilized the mRNA. We could not confirm the lack of ATP13A2 protein due to the lack of specific antibodies against the rat ATP13A2 protein. This homozygous gene KO was not embryonically lethal, and pups appeared to be normal at birth.

### Neurodevelopmental impact of Atp13a2 KO

**Fig. 2:**
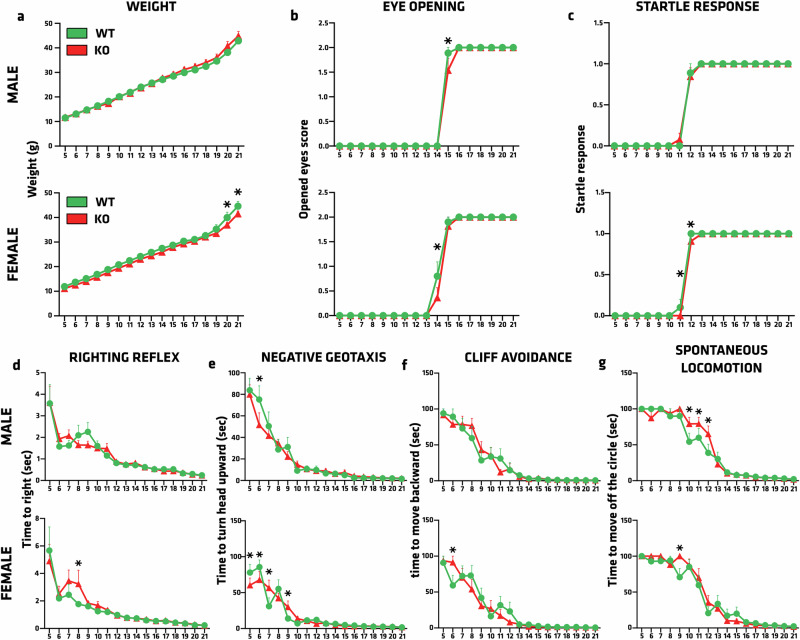
Assessment of neurodevelopmental milestones in *Atp13a2* KO rat pups shows some differences, primarily in female pups. Animals were tested daily between postnatal days 5 and 21. The following parameters were assessed: **a** body weight, **b** eye-opening, scored as 0 = both eyes closed, 1 = one eye open, or 2 = both eyes open; **c** startle reflex; **d** righting reflex measured as the time taken to flip/roll over onto its four paws, **e** negative geotaxis recorded as the time taken by the pup to turn 180° with the head upward. **f** Cliff avoidance is recorded as the time taken by the pup to move backward on the surface. **g** Spontaneous locomotion recorded as the time taken by the pup to move over a fixed distance. Data are expressed as mean ±SEM, *n* = 9–13 per group. *p < 0.05, Tukey’s post hoc test following a significant two-way ANOVA.

KRS is a juvenile-onset disease. Thus, neurodevelopment was assessed in *Atp13a2* WT and KO rats from postnatal days (P) 5 to 21 (Fig. 2). Body weight was similar between male WT and KO pups. Conversely, KO females weighed less than WT females from P20 onwards (Fig. 2a), which suggested a putative effect of the deletion on metabolism or energy expenditure in females. Strikingly, the ability to open the eyes (Fig. 2b), an important developmental milestone, was 1 day delayed in both male and female KO rats compared to WT controls at P14 for females (*p* < 0.0001) and P15 for males (*p* < 0.0001). Nevertheless, all animals had both eyes open at P16 regardless of genotype. The acoustic startle response was delayed two days in females at P11 (*p* = 0.0024) and P12 (*p* = 0.0058), but still not in males (Fig. 2c). These results implied a limited but significant delay in the neurodevelopment of ATP13A2-depleted rats. Since PD and KRS are mainly associated with motor deficits such as akinesia or bradykinesia, we assessed locomotor development in four different experiments during the neonatal period (Fig. 2d–g). First, the righting reflex, i.e., the capacity for the pup to flip over onto its four paws once placed on its back (Fig. 2d), revealed a significant slowing in females at P8 (*p* = 0.0287). Negative geotaxis, also testing sensory and proprioceptive functions, displayed occasional significant differences between WT and KO male pups at P6 (*p* = 0.002), and between WT and KO female pups from P5 to P7, and at P9 (0.0012< *p* < 0.0411) (Fig. 2e). In the same way as the righting reflex, the cliff avoidance test was well performed by the animals, except at P6 in females (*p* = 0.0033) (Fig. 2f). The measure of spontaneous locomotion gave a glimpse of a critical period for motor development in this model between P9 and P12 (Fig. 2g), with an increased time to move outside the experimental circle in KO pups. These neurodevelopmental features allowed us to better understand the early life of *Atp13a2* KO rats, showing mild developmental delays with a critical locomotor period.

### Motor behavior is affected in adult ATP13A2 KO rats

**Fig. 3:**
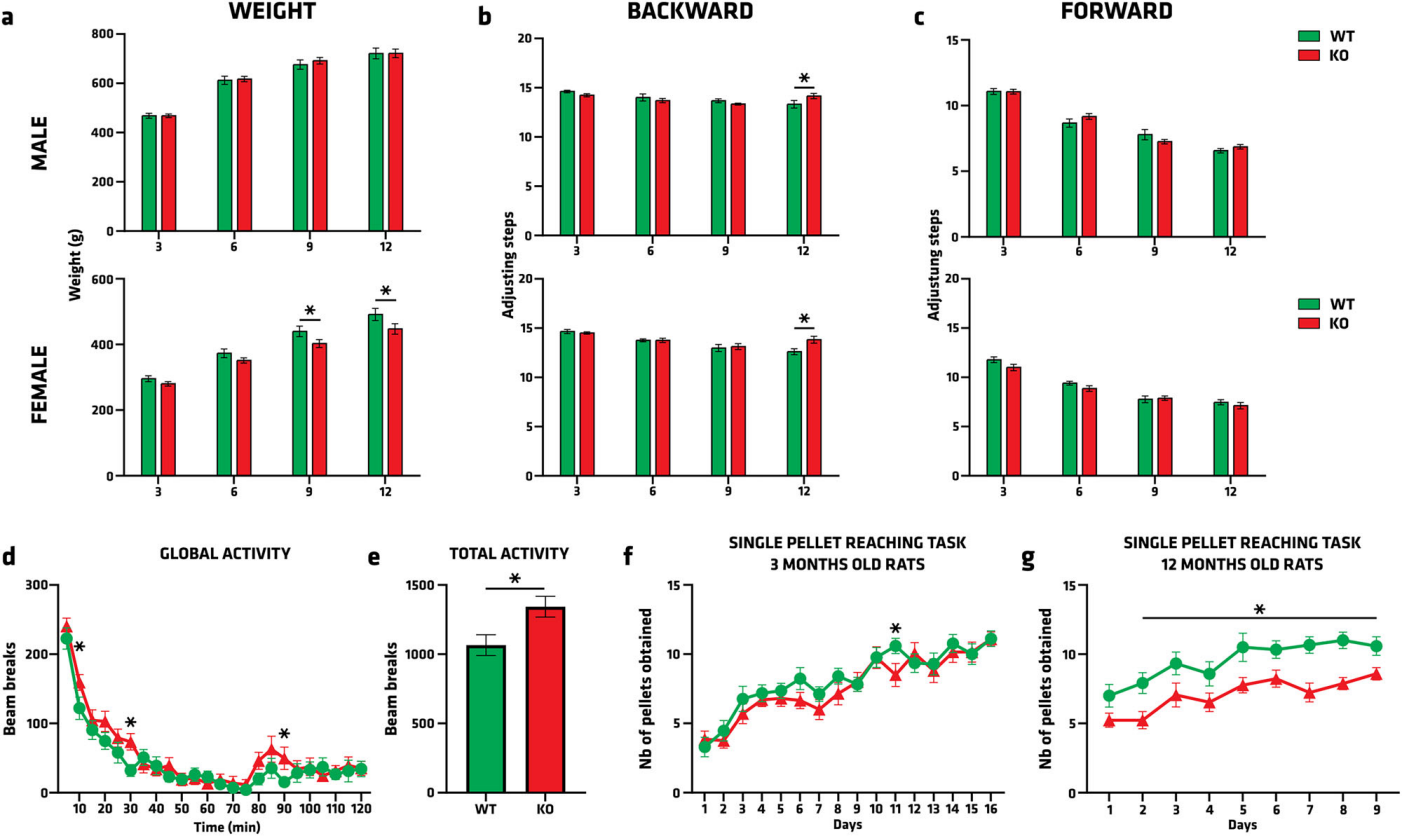
Motor behavioral assessments with the single-pellet reaching task and actimetry tests reveal significant motor affection in ATP13A2 KO rats. **a** Body weight was measured after 3, 6, 9, and 12 months of age. **b**–**e** The stepping test was measured by looking at **b** backward and **c** forward, adjusting steps. Analysis of the **d** global and **e** total locomotion was carried out over two hours, measuring the beam breaks over 5-minute intervals. **f**, **g** Reaching success (number of pellets obtained out of 20) by WT and *Atp13a2* KO rats and rats in the single-pellet task at **f** 3 months and **g** 12 months, evaluated over 15 and 9 days, respectively. Data are expressed as mean ±EM, *n* = 15–19 per group. *p < 0.05, Tukey’s post-hoc test following a significant two-way ANOVA, and unpaired Student’s *t* test when appropriate.

Following neonatal characterization of the *Atp13a2* KO rat strain, motor and fine motor skills were evaluated between 3 and 12 months old (mo) (Fig. 3). After the initial weight observations in newborns, the lower average body weight in KO females compared to control animals remained significantly decreased after 9 months of life (9mo: *p* = 0.0468; 12mo: *p* = 0.0188) (Fig. 3a), strengthening possible metabolic or energy expenditure issues caused by ATP13A2 dysfunction in the females. Regarding locomotor assessment with the stepping test (Fig. 3b, c), we observed significant differences at 12mo where KO animals performed more backward adjusting steps in the session compared to WT rats (Fig. 3b). Conversely, no differences between groups were observed for forward adjusting steps (Fig. 3c). The global activity, recorded in the open field over two hours, showed higher activity of KO rats at 10 min (*p* = 0.0231), 30 min (*p* = 0.0116), and 90 min (*p* = 0.0363) (Fig. 3d). Collectively, these motor activity results demonstrated hyperactivity in KO rats compared to controls, with 1065 beam breaks in WT vs. 1342 in KO (*p* = 0.0143) (Fig. 3e), a symptom recently described in a KRS patient^34^. Finally, as KRS is part of the Parkinsonism spectrum, assessing fine motor skills remains an essential validation for this experimental animal model. Herein, we used a single pellet reaching test conducted over 15 days, at 3 (Fig. 3f) and 12mo (Fig. 3g). At 3mo, KO rats were as efficient as WT, except at day 11 when they obtained fewer pellets (*p* = 0.0431). More interestingly, the single pellet reaching task at 12mo resulted in a significantly lower number of pellets obtained by KO rats from the 2nd day of the experiment (Genotype effect *p* < 0.0001; Time effect *p* < 0.0001; P2-P9: 0.0005 < *p* < 0.0411) These results suggested a fine motor skills deficit in 12-month-old rats, and provided an interesting behavioral readout in KRS and PD modeling.

### Pathological and Neurochemical phenotyping of Atp13a2 KO rats

**Fig. 4:**
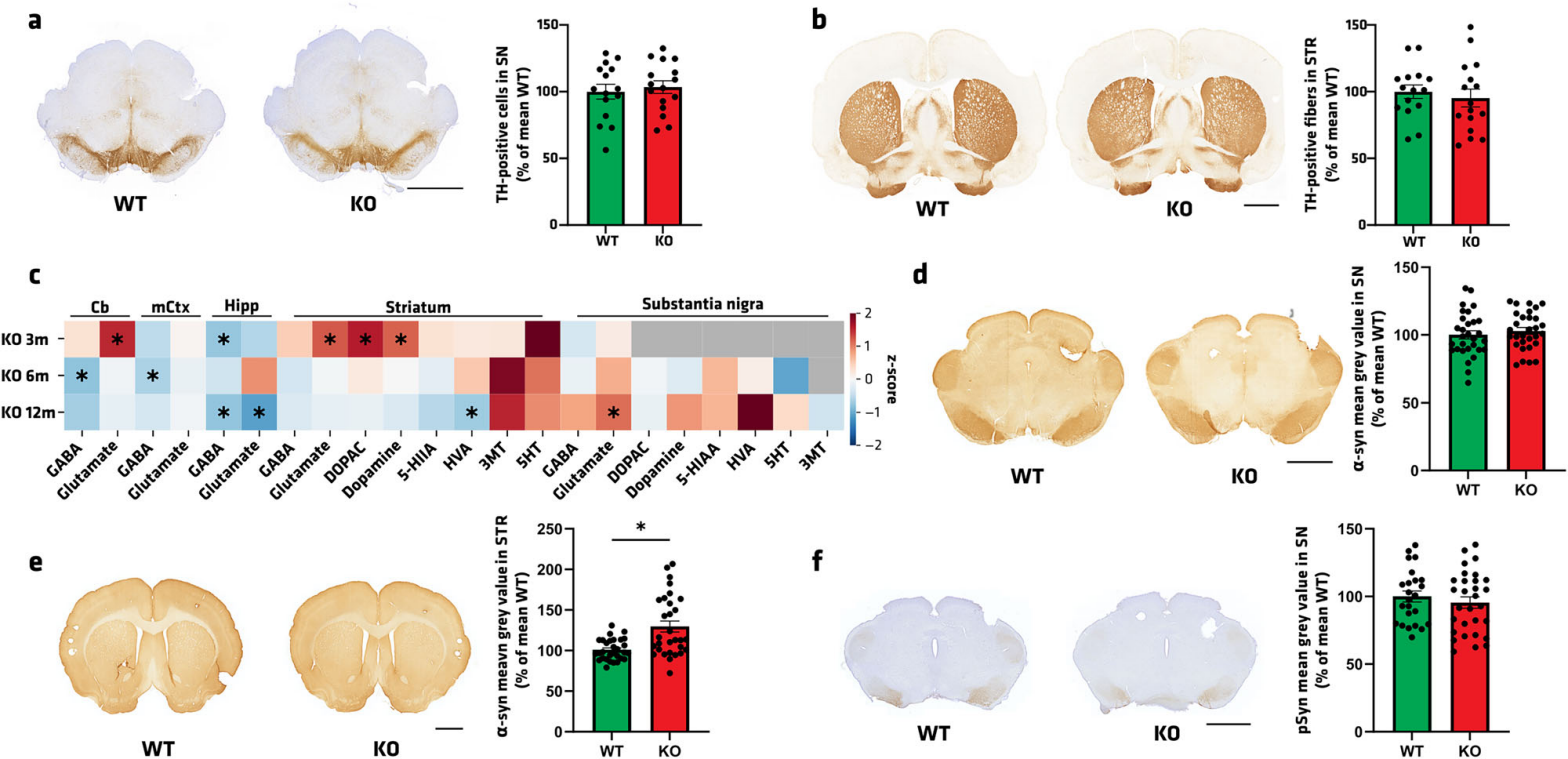
Nigrostriatal integrity is preserved with neurotransmitter modulation in *Atp13a2* KO rats. **a**, **b** Illustrative images and quantification of TH-immunohistostaining **a** stereological count in SN and **b** mean gray value in the striatum of 16-month-old rats. **c** HPLC analysis of GABA, glutamate, DOPAC, dopamine, 5-HIAA, HVA, 3MT, and 5-HT in the cerebellum (Cb), motor cortex (mCtx), hippocampus (Hipp), striatum, and substantia nigra in 3-, 6-, and 12-month-old KO rats compared to WT. **d**–**f** Illustrative images and quantification of endogenous α-syn immunostaining in **d** SN and **e** STR. **f** Illustrative images and quantification of pS129-α-syn immunostaining in the SN. Data are expressed as mean ±SEM, or z-score, *n* = 15–19 per group. *p < 0.05, unpaired Student’s *t* test. Scale bar = 2 mm.

Despite the subtle differences at the behavioral level, we explored putative PD-related pathological characteristics induced by the *Atp13a2* gene depletion in rats. First, we investigated the integrity of the dopaminergic nigrostriatal pathway in the oldest cohort of rats (i.e., 16-month-old) to observe potential disease-relevant lesions. Patients affected by PD and KRS show nigral dopaminergic neurodegeneration with striatal dopaminergic axon terminals^4,35–37^. TH-staining analysis in the SN revealed that loss of ATP13A2 is not sufficient to induce dopaminergic neuronal death (Fig. 4a). Accordingly, striatal TH staining showed no significant striatal dopaminergic terminal loss in KO compared to WT rats (Fig. 4b). Because changes in dopaminergic neurotransmission can precede neurodegeneration, we also examined the content of neurotransmitters and their metabolites by high-performance liquid chromatography (HPLC) in 5 different brain structures extracted at ages 3, 6, and 12mo in KO and WT rats (Fig. 4c). All data were normalized with the controls of the same age. We found age-dependent abnormalities in basal levels of one or more neurotransmitters in multiple brain regions in *Atp13a2* KO rats. In the cerebellum, we observed elevated levels of glutamate at 3mo (*p* = 0.027) and reduced levels of GABA at 6mo (*p* = 0.013), indicating possible granule and Purkinje cell dysfunctions. We found mild differences in GABA and Glutamate levels in the motor cortex. A significant reduction in both GABA (*p* = 0.0093) and glutamate (*p* = 0.036) was detected in the hippocampus at 12mo. In the nigrostriatal pathway, a significant increase in striatal content of glutamate (*p* = 0.018), dopamine (*p* = 0.0072), and 3,4-dihydroxyphenylacetic acid (DOPAC) (*p* = 0.0061) was found at 3mo, while homovanillic acid (HVA) was significantly decreased at 12mo (*p* = 0.045). Glutamate also showed an age-dependent dysregulation in the SN, with a significant increase at 12mo (*p* = 0.047). Overall, *Atp13a2* KO rats displayed age- and region-specific neurotransmitter changes, which could indicate compensatory adaptations.

Then, we set out to quantify the expression levels of total α-syn and α-syn phosphorylated at serine 129 (pSyn) as a proxy of the pathogenic form of α-syn. First, total endogenous α-syn immunolabeling in the SN of 16-month-old rats remained similar between genotypes (Fig. 4d). At the same time, a significant increase in α-syn staining was observed in the striatum of KO animals (Fig. 4e), suggesting an accumulation of synaptic α-syn in this structure. Second, focusing on the SN, no genotype difference was observed in pSyn immunolabeling (Fig. 4f). These results showed that loss of ATP13A2 does not lead to significant dopaminergic neurodegeneration but to mild neurochemical and α-syn pathological alterations.

### Neuroinflammation induced by ATP13A2 loss of function

As PD patients have significantly higher levels of inflammation in several brain regions than non-affected individuals, we evaluated the impact of genotype on inflammation. We performed histological analysis of microglial proliferation through Iba1 staining (Supplementary Fig. 1) and astrocytic reaction through GFAP staining at 16mo (Supplementary Fig. 2). Microglia surface staining in the striatum (Supplementary Fig. 1a), the SN (Supplementary Fig. 1b), and the cortex (Supplementary Fig. 1c) of KO rats did not differ from controls. However, a significant increase in the microglial surface in the hippocampus (*p* = 0.0323) was observed in KO rats, mainly in females (*p* = 0.0363, Supplementary Fig. 1d). Conversely, the astrocytic reaction to ATP13A2 depletion was more intense in the striatum (*p* = 0.0097, Supplementary Fig. 2a), SN (*p* = 0.0303, Supplementary Fig. 2b), and motor cortex (*p* = 0.0093, Supplementary Fig. 2c) of KO rats than WT rats. Sex stratification revealed significantly higher intensity in the male compared to female striatum (*p* = 0.0207) and cortex (*p* = 0.0254), whereas the opposite was true for the SN (*p* = 0.046). These data suggested that neuroinflammation is induced in a sex- and region-specific manner in *Atp13a2* KO rats, where the striatum, SN, and cortex were linked to an astrocytic reaction and the hippocampus to a microglial response.

### The autophagy process is defective in the SN of ATP13A2 KO rats

**Fig. 5:**
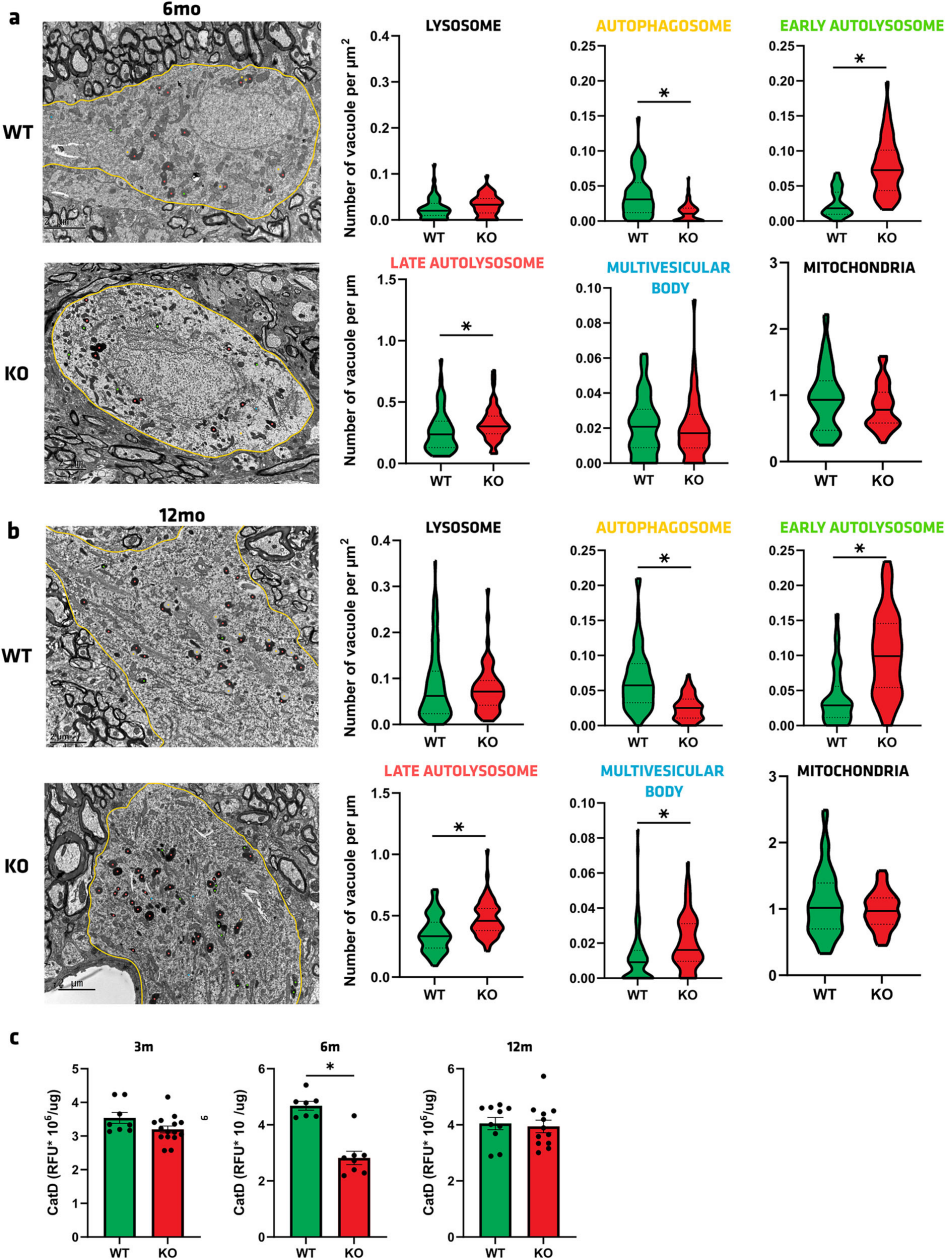
Accumulation of lysosomal-related structures in *Atp13a2* KO. **a**, **b** Quantification of the different autophagy-related vacuoles (lysosome, autophagosome [yellow], early- [green] and late-autophagosome [red], multivesicular body [blue]) and mitochondria under electron microscopy in dopaminergic neurons of the SN in **a** 6- and **b** 12-month-old WT and KO rats (*n* = 3–5 per group). Scale bar = 2 µm. **c** CatD activity in purified lysosomal fractions from midbrain tissues at 3-, 6-, and 12-month-old WT and KO rats. Data are expressed as mean ±SEM. *p < 0.05, unpaired Student’s *t* test.

ATP13A2 is a lysosomal polyamine-transporting ATPase, implicated in the regulation of luminal pH of lysosomes, the central organelle in the ALP. Its dysfunction is linked to a luminal lysosomal alkalinized pH^12^ and enhanced sensitivity to heavy metals^38–43^. ALP is dysregulated in KRS models^12,13,44^. To determine whether loss of ATP13A2 may underlie PD-linked lysosomal alterations, we first quantified the density of lysosomes, autophagosomes, early- and late-autolysosomes, multivesicular bodies, and mitochondria in dopaminergic neurons of the SN of 6- (Fig. 5a) and 12mo rats (Fig. 5b), through electron microscopy examination. The number of lysosomes was similar in WT and KO rats at both ages. Similar results were obtained by immunoblot of the total lysosomal-associated membrane protein (LAMP)-2 protein levels (Supplementary Fig. 3a) and in lysosomal-enriched fractions from midbrain tissues (Supplementary Fig. 3b). To verify the expression of the Vacuolar-type proton (H^+^) ATPase (V-ATPase) between WT and KO rats, we performed immunoblots in nigral and striatal protein samples and found that the levels of ATP6V1A were unaffected by the genotype (Supplementary Fig. 3c, d). Interestingly, a significant decrease in autophagosomes from 0.0386 per µm^2^ in WT to 0.01161 per µm^2^ in KO rats was noticed at 6mo (*p* < 0.0001, Fig. 5a), and at 12mo, with a reduction in KO by a factor of 2.5 (*p* < 0.0001, Fig. 5b). The opposite was found for the early and the late autolysosomes, where an increase in vacuoles per µm^2^ was observed at 6 and 12mo (*p* = 0.0338 for late autolysosomes at 6mo, *p* < 0.0001 for the rest). This accumulation of autolysosomes containing intact or partly degraded cytoplasmic material could be attributed to a lysosomal pH deficiency caused by ATP13A2 depletion, which impedes content degradation and ultimately leads to vacuole accretion. The number of multivesicular bodies, i.e., endocytic organelles recognizable by their abundant luminal vesicles, was similar between KO (0.08976 per µm^2^) and WT (0.09647 per µm^2^) at 6mo (*p* = 0.6682). Instead, a significant 1.76-fold increase in KO rats was measured at 12mo (*p* = 0.0003, Fig. 5b), reinforcing previous results of vacuole accumulation in ATP13A2-depleted animals. Mitochondria morphology and density were also examined to determine if genotype induced a putative bioenergetics compartment deficit. No significant difference was observed between genotypes at 6 and 12mo.

To corroborate the view of impaired lysosomal-mediated degradation in KO rats, as evidenced by the abundance of autolysosomes, we next performed in vitro assays of CatD activity in purified lysosomal-enriched fractions from midbrain tissues, which confirmed a markedly reduced proteolytic activity at 6mo (Fig. 5c), indicative of an impaired cathepsin maturation process.

**Fig. 6:**
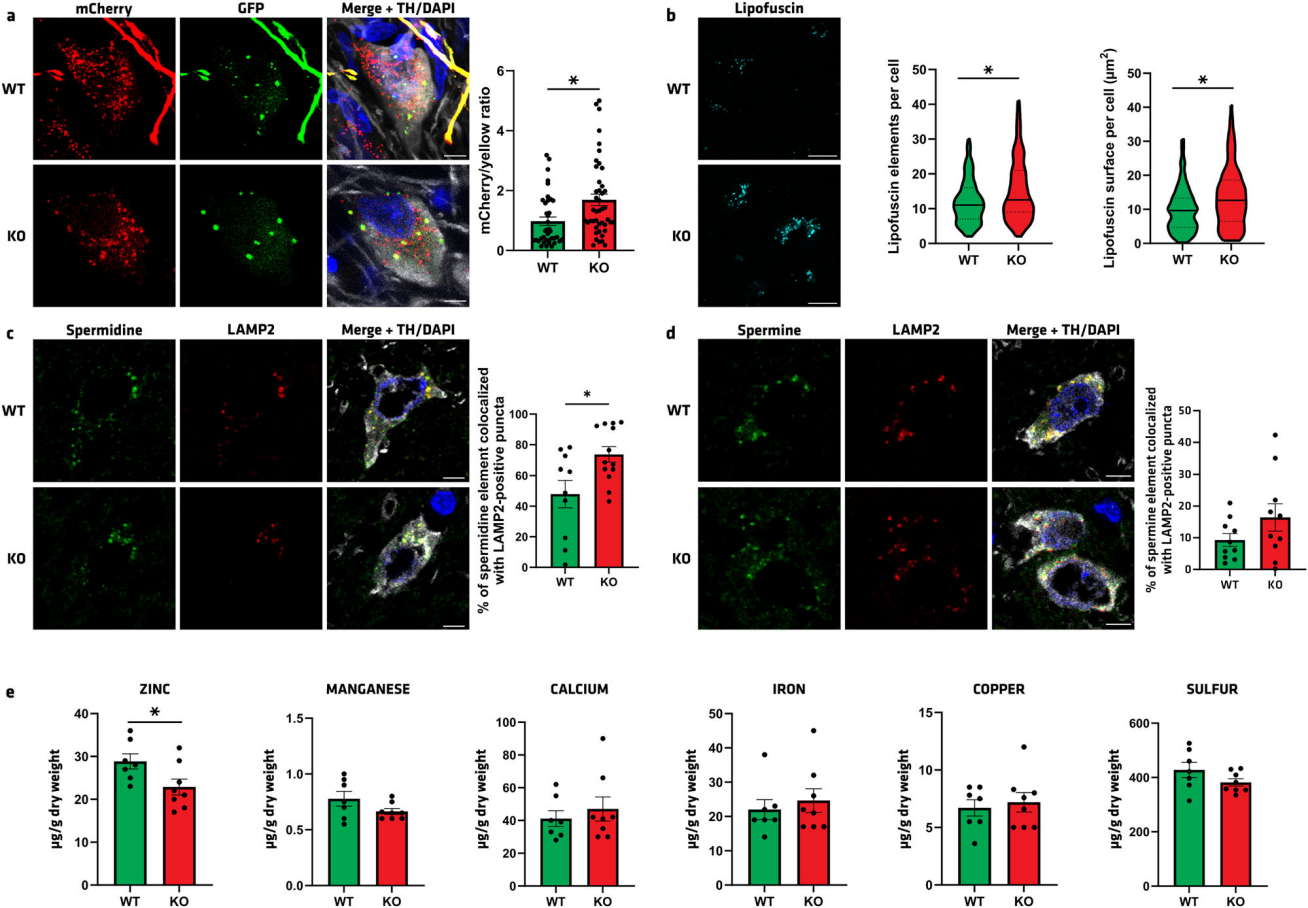
Autophagy flux, lysosomal polyamines, and heavy metal homeostasis are perturbed in *Atp13a2* KO rats. **a** Illustrative imaging and quantification in the SN of mCherry/GFP puncta after nigral injection of AAV2/9-mCherry-GFP-LC3 reporter in 12-month WT and KO rats. **b** Illustrative images and quantification of lipofuscin dots per cell in the SN of 16-month-old rats. **c**, **d** Illustrative imaging and quantification of **c** spermidine- and **d** spermine-positive elements colocalized with LAMP2 puncta in TH-positive neurons of the SN in 16-month WT and KO rats. **e** Levels of zinc, manganese, calcium, iron, copper, and sulfur measured by synchrotron X-ray fluorescence in the SN of WT and ATP13A2 KO rats (red) at 12 months of age (*n* = 7–8). Data are expressed as mean ±SEM. *p < 0.05, unpaired Student’s *t* test. Scale bar **a**, **c**, **d** = 5 µm, **b** = 20 µm.

Further supporting the occurrence of an accumulation of autolysosomes in *Atp13a2* KO animals, we injected an AAV pseudotype 2/9 containing CMVp-mCherry-GFP-LC3 vector in the SN^45^. This virus expresses a tandem fluorophore with acidic-sensitive GFP linked to the autophagosomal-related protein LC3, allowing the measurement of ALP activity in vivo^46^. The GFP fluorescent signal of the reporter is sensitive to acidic conditions; thus, the colocalization of green and red fluorescence (yellow puncta) indicates that the tandem protein is not localized in compartments fused with a lysosome, while the detection of red puncta indicates that the protein is located in the autolysosome. Confocal analysis showed that the ratio between the number of mCherry and yellow dots, taken as an index of the LC3 flux, was significantly elevated, suggesting an overall increase of mCherry dots in *Atp13a2* KO rats at age 12mo (*p* = 0.0047, Fig. 6a). Overall, the data suggest that autophagolysosomes are accumulating, but their degradation with lysosomes is impaired. These results pointed to a blockage in autophagy flux, where autophagosomes are being generated, but the degradation step is either delayed or defective. This can indicate a functional defect in autophagy, possibly due to issues with autophagosome-lysosome fusion, lysosomal dysfunction, or inhibition of the autophagic process.

KRS is linked to autophagy dysfunction, and it is well-known that one pathological hallmark is the presence of lipofuscin in patients and in different ATP13A2-related animal models^15,31,32,39,47^. We then measured the number and surface of lipofuscin deposits in the SN (Fig. 6b). Confocal observation revealed increased lipofuscin elements per cell from 11.95 in WT to 15.15 in KO rats (*p* = 0.0005, Fig. 6b left). This was associated with the increased lipofuscin-occupied surface in KO compared to WT rats (13.53 µm^2^ vs. 9.915 µm^2^, respectively, *p* = 0.0002, Fig. 6b right). Moreover, polyamine-positive vesicles colocalized with LAMP2-positive puncta in dopaminergic neurons of the SN were measured (Fig. 6c, d). In KO rats, spermidine-positive vesicles are significantly more colocalized in lysosomes (*p* = 0.0144, Fig. 6c), and spermine tends to be more retained in the lysosomal lumen compared to the WT group (*p* = 0.1511, Fig. 6d). These results indicate that ATP13A2 deficiency results in the accumulation of polyamines within lysosomes, consistent with a defect in their export from the lysosomal lumen. Finally, heavy metals quantification in the SN through synchrotron X-ray fluorescence analysis revealed a significant decrease in zinc (*p* = 0.0375) but not manganese, calcium, iron, copper, and sulfur concentrations in the SN of KO rats (Fig. 6e), reminiscent of a phenotype described in the literature^48^. Overall, these results indicated autophagy dysregulation with a decrease in autophagosome number and an accumulation of autolysosomes, associated with zinc dyshomeostasis and lipofuscin deposits in *Atp13a2* KO rats at 12 months.

### Electrophysiological alterations of SN dopaminergic neurons

**Fig. 7:**
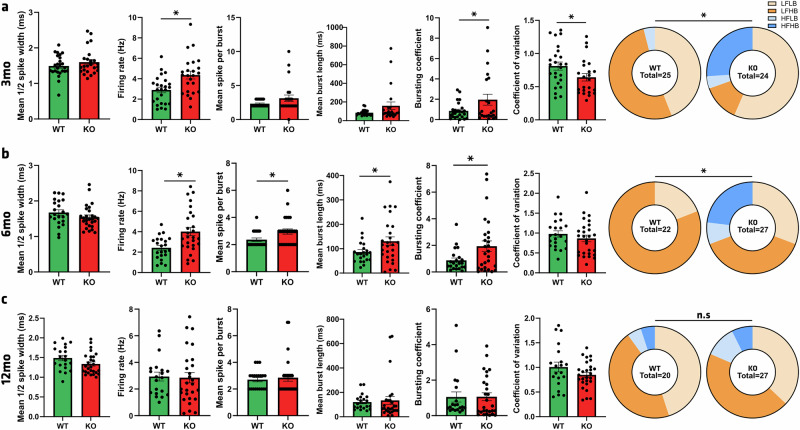
Electrophysiological analysis shows bursty dopaminergic neurons of the SN in 3-, 6-, but not 12-month-old *Atp13a2* KO rats. Electrophysiological record of the mean ½ spike amplitude, firing rate, mean spike per burst, mean burst length, bursting coefficient, coefficient of variation, and categorical affiliation (LFLB low firing low bursting, LFHB low firing high bursting, HFLB high firing low bursting, HFHB high firing high bursting) in WT and KO rats at **a** 3-, **b** 6-, and **c** 12-month old. Data are expressed as mean ±SEM. *p < 0.05, unpaired Student’s *t* test or Chi-square test when appropriate.

Even though no overt neurodegeneration was observed, multiple factors of cellular suffering were identified, such as inflammation and autophagy impairment. We aimed to characterize electrophysiological parameters of dopaminergic neurons of the SN in 3-, 6-, and 12-month-old rats (Fig. 7). Interestingly, at 3mo an increase in the dopaminergic neuron firing rate was recorded in KO rats (4.36 ± 0.38 Hz) compared to WT (2.89 ± 0.27 Hz) (*p* = 0.0026, Fig. 7a). It also appeared that dopaminergic neurons in *Atp13a2* KO rats were more bursty, with a 2.3-fold bursting coefficient elevation compared to WT rats (*p* = 0.0446, Fig. 7a). These results transcribe a modulation in the 4 main categories used to classify dopaminergic cells of the SN, with an important proportion (25%) of high-firing, high-bursting neurons recorded in KO rats, which are completely absent in the WT group (*p* = 0.0056). Three months later (Fig. 7b), we found the same significant differences in firing rate (*p* = 0.0024) and bursting coefficient (*p* = 0.0282), to which was added a significant increase of the mean spike number per burst (*p* = 0.018), and concurrently a mean burst length stretched to 130.5 ± 17.90 ms per burst in KO compared to 86.17 ± 9.96 ms in WT animals (*p* = 0.0476), still representing a divergence in dopaminergic population (*p* = 0.0139). These significant modifications suggested that burstiness caused by ATP13A2 depletion returns to normal at 12mo, with a clear decrease in the number of high-firing, high-bursting neurons (Fig. 7c). Finally, *Atp13a2* gene depletion caused overactivation of dopaminergic neurons in the SN, making them more bursty than in control animals, but only up to 6mo, with a return to physiological electrophysiological parameters at 12mo.

### Atp13a2 KO does not worsen AAV-A53T-induced pathology

**Fig. 8:**
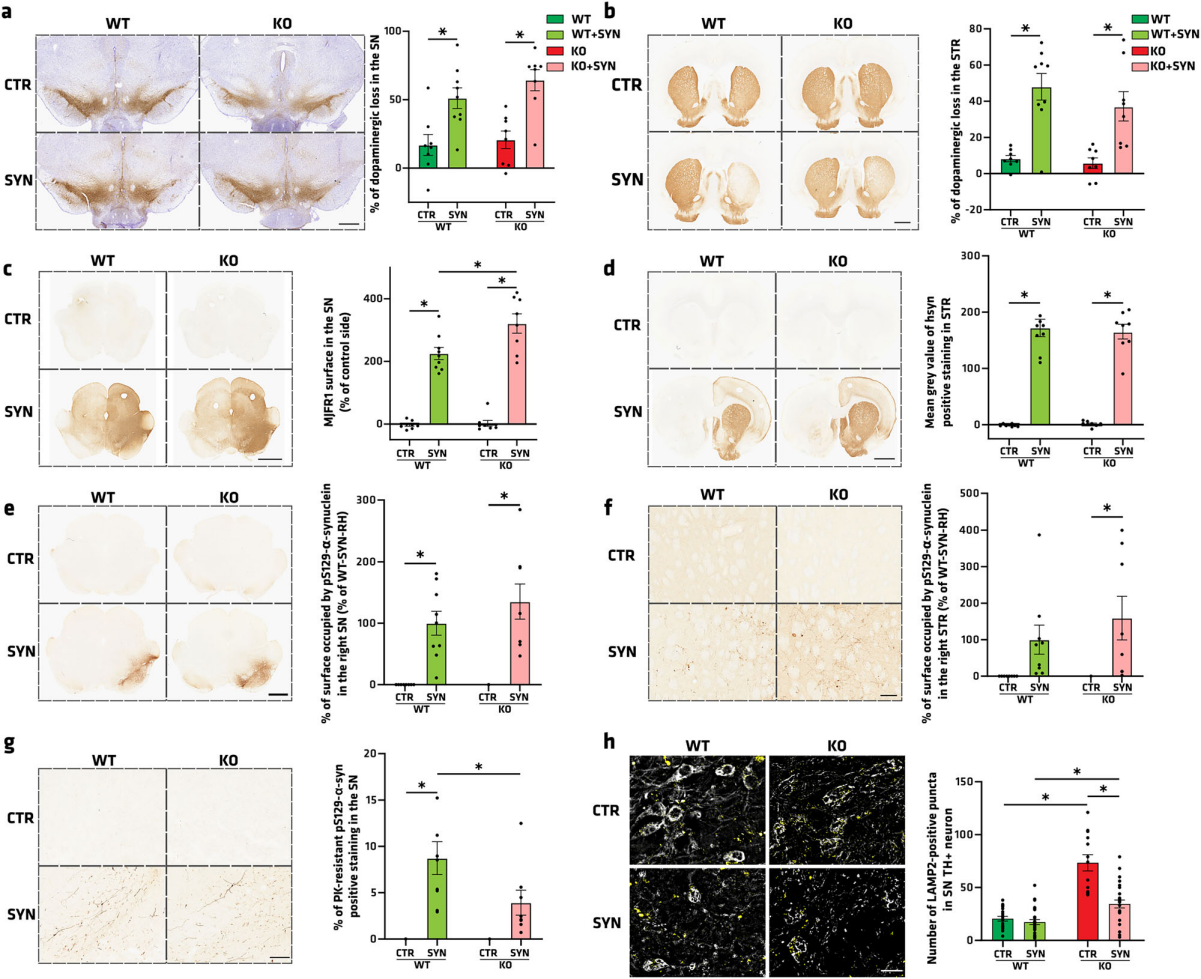
*Atp13a2* KO does not worsen AAV-A53Tα-syn-induced pathology but affects ALP. **a** Illustrative images and quantification of TH-positive neurons using stereology in the SN of WT rats injected with SYN vector (WT + SYN) or Stuffer vector (WT-CTR), and KO rats injected with SYN vector (KO + SYN) or Stuffer vector (KO-CTR). **b** Illustrative images and quantification of TH-positive fibers in the striatum through mean gray value analysis. **c**, **d** Illustrative images and quantification of human α-syn-positive immunostaining in **c** the SN and **d** the striatum of WT and KO rats injected with SYN vector or Stuffer vector. **e**, **f** Illustrative images and quantification of S129 phosphorylated α-syn (pSyn) positive immunostaining in **e** the SN or **f** the striatum of WT and KO rats injected with SYN vector or Stuffer vector. **g** Illustrative images and quantification of pSyn-positive immunostaining after proteinase K (PK) treatment in the SN of WT and KO rats injected with SYN vector or Stuffer vector. **h** Illustrative images and quantification of LAMP2 (yellow)-positive immunofluorescent staining in TH (gray)-positive cells in the SN of WT and KO rats injected with SYN vector or Stuffer vector. Data are expressed as mean ±SEM.*p < 0.05, Tukey’s post-hoc test following a significant two-way ANOVA. Scale bar **a**–**e** = 4 mm, **f**, **g** = 100 µm, **h** = 20 µm.

To determine whether the loss of ATP13A2 and its impact on ALP function in the SN and on the activity of dopaminergic neurons could increase the vulnerability of dopaminergic neurons to PD pathology, 3mo rats received an unilateral intranigral injection of either an AAV2/9 carrying human A53T α-syn (pAAV2-CMVie/hSyn-synA53T-WPRE-pA) (SYN) to induce nigrostriatal neurodegeneration, as previously reported in rats^49–52^ or a control AAV2/9 “AAV-stuffer” (pAAV2-CMVie/hSyn-WPRE-pA) (CTR). Five months after injection, stuffer-injected WT rats displayed approximately 16.88% loss compared to 20.65% in KO rats, while we measured a 51.14% neurodegeneration compared to the control side in WT animals injected with the AAV-A53T (WT + SYN) and 64.31% in the KO + SYN group, as determined by stereological counting of SN TH-positive cells (Fig. 8a). Similar results were obtained in the striatum, with a significant reduction of dopaminergic fibers in SYN cohorts compared to CTR animals (Fig. 8b). These results indicated that the genotype does not significantly exacerbate α-syn-induced dopaminergic neurodegeneration.

We next attempted to examine the human-specific α-syn (hα-syn) pattern in SN and striatum. We observed a significantly higher level of hα-syn in KO + SYN than in WT + SYN in the SN (Fig. 8c). No difference was observed in the hα-syn level in the striatum (Fig. 8d). Finally, we explored α-syn pathology with pSyn antibody. However, although the SYN groups show pSyn at the SN level compared to the CTR groups, no significant difference was noticed between the genotypes in the SYN condition (Fig. 8e). Moreover, we observed the presence of pSyn-positive staining only in the striatum in the KO-SYN group compared to the KO-CTR group (*p* = 0.0048) (Fig. 8f). Then, we examined the effects of α-syn overexpression on α-syn species identified by a pS129-α-syn antibody in the absence or presence of proteinase K (PK) pretreatment as a second indicator of pathological α-syn (Fig. 8g). Surprisingly, PK treatment reveals that pSyn staining in KO-SYN rats is more sensitive than in WT-SYN, where 8.74% of the pSyn is PK resistant (*p* = 0.0372, Fig. 8g). These results prompted us to further investigate the impact of downregulating ATP13A2 levels on human α-syn preformed fibril-induced mouse α-syn expression and phosphorylation in primary mouse mesencephalic cultures (Supplementary Fig. 4). The lack of potentiating effect in this in vitro context confirmed the absence of potentiation in decreasing ATP13A2 levels for increased expression of pSyn (Supplementary Fig. 4).

In α-syn injected rats, we also looked at the microglia and we observed an enhanced microglial activation in the SN of KO-SYN compared with WT-SYN (*p* = 0.0059) and KO-CTR (0.0188, Supplementary Fig. 5). Even if the differences between WT and KO groups remained subtle after α-syn-induced dopaminergic neurodegeneration, *Atp13a2* depletion accentuated hα-syn protein expression level (Fig. 8c), confirming and extending the proposal that the observed accumulation of hα-syn is potentially due to autophagy-related alterations in KO rats. Finally, we explored whether autophagy was more affected in the SYN groups by using the lysosomal-related marker LAMP2 through immunofluorescence analysis. We found more LAMP2-positive puncta in SN dopaminergic neurons in *Atp13a2* KO animals, regardless of whether they had been injected with SYN or CTR. However, SYN injection showed a significant increase to 73.38 LAMP2-positive puncta per cell, compared with 34.46 in the KO-CTR condition (*p* < 0.0001) (Fig. 8h). Finally, it is interesting to notice that ATP13A2 depletion had no impact on the neurodegenerative process in the SN and the striatum in this A53T rat model, even though SN hsyn and STR pSyn levels were higher in the KO + SYN condition, which might counterbalance the less PK-resistant nature of pSyn.

### AAV-hTyrosinase-induced pathology is not exacerbated by Atp13a2 depletion

**Fig. 9:**
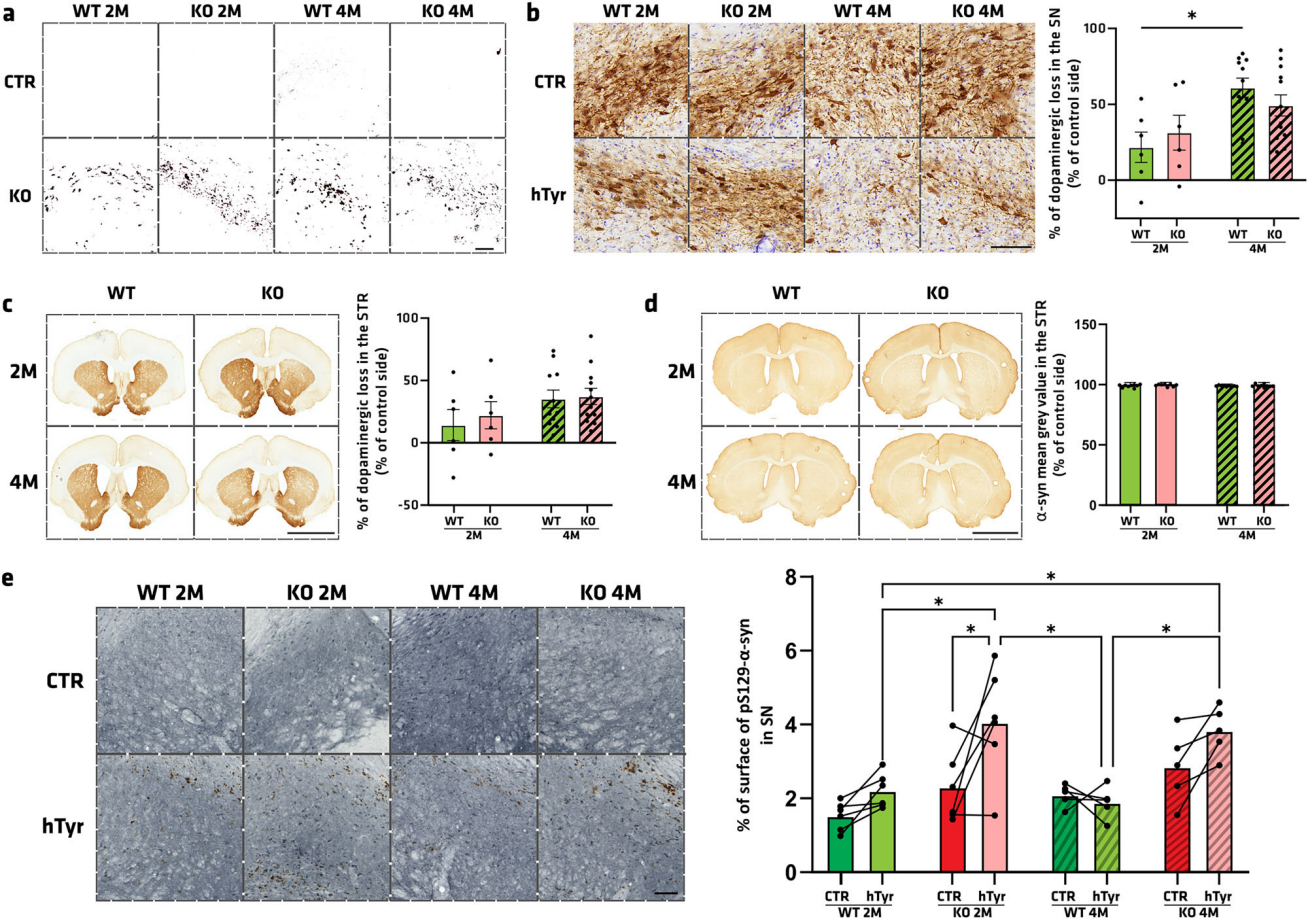
AAV-hTyrosinase-induced pathology is not exacerbated by *Atp13a2* depletion. **a** Observation after Fontana staining of neuromelanin and threshold post-treatment (black) in the SN of WT and KO animals on the side injected with tyrosinase vector (hTyr) or the side injected with Stuffer vector (CTR), 2 and 6 months after surgery. **b** Illustrative images and quantification of TH-positive neurons using stereology in the SN of rats injected with hTyr 2 or 4 months post-surgery. **c** Illustrative images and quantification of TH-positive fibers in the striatum through mean gray value analysis. **d** Illustrative images and quantification of human α-syn-positive immunostaining in the striatum. **e** Illustrative images and surface quantification of pSyn-positive signal in the SN revealed by VectorSG staining. Data are expressed as mean ±SEM.*p < 0.05, Tukey’s post-hoc test following a significant two-way ANOVA. Scale bar **a**, **b** = 100 µm, **c**, **d** = 4 mm, **e** = 100 µm.

To investigate the genotype susceptibility to PD, we tested the consequences of expressing human tyrosinase in the SN, aiming to reproduce the age-dependent human-like neuromelanin accumulation within nigral dopaminergic neurons^53^. We unilaterally injected 2 µL of an AAV-hTyr in the SN to induce neuromelanin-related pathology (hTyr) and an AAV-Stuffer (8.52 × 10^12^ vg/ml) as the control in the contralateral SN (CTR). Analysis was carried out at 2 and 4 months after surgery to follow the progression. To validate that AAV-hTyr allowed expression of the transgene in the SN, neuromelanin was counterstained with the melanin marker Masson-Fontana. In both genotypes, we revealed black dots accumulated in the SN on the hTyr-injected side (Fig. 9a). In contrast, the contralateral SN from the same animals remained unstained, as rodents lack neuromelanin. Classical measurements of nigrostriatal integrity were performed through stereological counts of TH-positive neurons in the SN compared to the CTR side. Two months after surgery, WT rats displayed a 21.7% loss of dopaminergic neurons, while KO rats 31.36%. This neurodegeneration worsened at 4 months post-injection, rising to 61.04% in WT rats and 49.33% cell loss in KO rats. However, while statistical analysis showed that neuron loss at 4 months was larger than at 2 months in WT rats (*p* = 0.021), no such difference was found in KO rats (Fig. 9b). In the striatum, we measured a dopaminergic loss of 14.23% at 2 months and 35.19% at 4 months in WT rats and 22.12% at 2 months and 37.23% at 4 months in KO rats (Fig. 9c), with no significant differences between the two time-points. The level of endogenous α-syn in the striatum remained stable across time, regardless of genotype (Fig. 9d). However, pS129 α-syn surface staining in the SN in the hemisphere injected with the AAV-hTyr appears to be significantly increased up to 4.05% in ATP13A2-depleted rats compared to the contralateral hemisphere and the control animals (Fig. 9e). To conclude, it is interesting to notice that genetic deletion of *Atp13a2* exacerbates phosphorylated α-synuclein levels in the SN, suggesting a vulnerability caused by the genotype in the pathogenic process and accumulation of pS129 α-syn.

## Discussion

Herein, we describe an *Atp13a2* KO rat model, enabling the study of Kufor-Rakeb Syndrome (KRS), an early-onset form of PD associated with impaired ALP functions. We included both sexes whenever possible to highlight sex differences, with careful attention to statistical analysis. This ATP13A2-null rat model was developed using CRISPR-Cas9-based deletion of the targeted genomic sequences, which leads to degradation of the *Atp13a2* mRNA production, and therefore suppresses protein expression. This new animal model reproduces some pathological features observed in KRS and idiopathic PD patients. First, it is important to notice a neurodevelopmental critical phase appearing a few days after birth, up to 2 weeks of life, which can be associated with the juvenile onset of symptoms in KRS patients, usually reported in adolescence, between the ages of 12 and 19 years^37,47,54,55^. It also appears that these neurodevelopmental differences observed in *Atp13a2* KO rats are sex-dependent, occurring more in females, suggesting a hormonal role in the development of this ATP13A2-related model. We then demonstrated an evolution of locomotor and fine motor symptoms at 12 months in this *Atp13a2* KO model, with the appearance of a hyperactivity phenotype, a symptom recently described in a KRS patient^34^, and fine motor skills deficits in the single-pellet reaching skill test. The latter makes it possible to study an important variable of KRS and PD, mainly known for its motor symptoms, which are described as rigidity, hypokinesia, and bradykinesia^36^. Nevertheless, this animal model shows no spontaneous neurodegeneration of the nigrostriatal dopaminergic pathway, neither in the SN nor in the striatum, even with advanced age ( ~ 16mo). This finding is important and confirms the presence of a compensatory mechanism in the germline ATP13A2-depleted animal model, as the adult induction of the ATP13A2 in mice^31^ and non-human primates^32^ recently provoked a nigrostriatal neurodegeneration. The absence of overt neurodegeneration in this constitutive *Atp13a2* KO model may reflect developmental compensation. One possibility is upregulation of related P5B-type ATPases, such as Atp13a3, which could mitigate polyamine dysregulation. Additionally, lysosomal and autophagy-related pathways may undergo early-life adaptive remodeling that preserves function in the long term. These compensatory processes could be less effective in adult-onset models, where sudden loss of Atp13a2 may lead to more pronounced neurodegeneration. This compensatory system is not understood and needs to be clarified in the future to better understand the developmental role of ATP13A2. Future studies examining transcriptional and proteomic changes in early postnatal brains may help identify such compensatory networks. In addition, no alterations in α-syn pathology were observed in SN, although a significant and mild accumulation of endogenous α-syn was observed in the striatum. These results are reminiscent of the phenotype of *Atp13a2* KO mice since these mice do not exhibit nigrostriatal pathway dopaminergic neurodegeneration or α-syn pathology up to 27 months of age, indicating that constitutive *Atp13a2* KO alone is insufficient to replicate PD-like neuropathology^30,56^, nicely reviewed in ref. ^15^.

Neurochemical phenotyping in different brain structures of *Atp13a2* KO rats revealed age-dependent alterations of one or more neurotransmitters, reminiscent of what has been observed in *PINK1*, *Parkin*, and *LRRK2* KO rats^57,58^. Although *Atp13a2*, *PINK1*, and *Parkin* KO rats all exhibit age-dependent motor phenotypes and mitochondrial/autophagic stress, their underlying mechanisms differ. Pink1 and Parkin are central to mitophagy, and their loss directly impairs mitochondrial turnover. In contrast, Atp13a2 regulates lysosomal polyamine export and homeostasis, with secondary impacts on mitochondrial function due to impaired autophagy and lysosomal dysfunction. Phenotypically, Atp13a2 KO rats show later-onset and more lysosome-centric pathology, including cerebellar involvement, compared to the early-onset dopaminergic deficits seen in Pink1 and Parkin models. From an inflammatory point of view, we showed reactive astrogliosis or microgliosis in a sex- and region-specific manner in *Atp13a2* KO rats, with the striatum, SN, and cortex being linked to an astrocytic reaction and the hippocampus to a microglial response. Reactive astrogliosis has also been reported in *Atp13a2* KO mice models^59^. We also show biochemical and cellular alterations relevant to KRS and PD in this rat model, with (i) a dysregulation in zinc in the SN, which is associated with many neurological disorders like PD^60–63^, and in ATP13A2-related models such as patient-derived human olfactory neurosphere cultures harboring *ATP13A2* loss-of-function mutations^40,43,48,64^; (ii) abnormalities in the endolysosomal pathway, such as a decrease of autophagosome elements accompanied by a significant accumulation of autolysosomes; and (iii) accumulation of polyamines in the lysosomal lumen due to ATP13A2 export role deficiency, already observed in ATP13A2 KO HAP1 cell line and siATP13A2 iPSC-neurons^65^. This lysosomal-related vesicle accumulation may be associated with the role of ATP13A2 in regulating the luminal acidic lysosomal pH, pathologically alkalinized when ATP13A2 is dysfunctional^12,44,66^ and, therefore, unable to degrade the autophagosome-delivered contents. This may lead to neuronal dysfunction, which was evidenced by an increase in bursting activity of SN dopaminergic neurons at 3 and 6 months. Notably, this electrophysiological alteration is observed in young, but not adult, ATP13A2-deficient animals, and is characterized by an increased firing rate and bursting activity. This may be attributed to the normal age-related decline in physiological firing of dopaminergic neurons^67–69^. ATP13A2 KO rats appear to amplify this age-dependent mechanism, exhibiting abnormally increased firing and bursting rates during early life, followed by a marked reduction in older age, ultimately returning to physiological levels observed in wild-type animals. Finally, *Atp13a2* deletion did not exacerbate or sensitize dopaminergic neurons to the neurotoxic effects of viral-based α-syn or human tyrosinase overexpression. This work highlights the molecular, neuropathological, and behavioral features of ATP13A2 loss-of-function within the rat life span, providing a useful animal model for studying how ATP13A2 depletion affects developmental processes and the ALP, and how endolysosomal dysfunction may contribute to neurodegeneration in PD and related neurodegenerative disorders.

Numerous other experimental models of ATP13A2 dysfunction have already been characterized in many species^15^. ATP13A2 knockdown was first performed in *Saccharomyces cerevisiae* with the deletion of *YPK9* (the yeast orthologue of *ATP13A2*)^10,70^ and then in fibroblasts or dopaminergic cell lines^8,12,44^, pointing out different sensitivity to divalent heavy metal ions and ALP alterations, such as alkalinized lysosomes with reduced activity. The first in vivo model was generated by knocking down the *ATP13A2* orthologue in *Caenorhabditis elegans*, confirming the protective role of ATP13A2 in manganese toxicity and showing an increase in misfolded α-syn^13,38^, which has also been replicated in a zebrafish model^71^. In 2013, Schultheis and colleagues reported an *Atp13a2* KO mouse model sharing multiple common characteristics with this rat model, such as lipofuscin accumulation and sensorimotor deficits without dopaminergic depletion^30,72^. In 2015, age-related motor dysfunction, reactive astrocytosis, ALP abnormality, and lipofuscin accumulation were reported in conditional ATP13A2 null mice obtained through the Cre/loxP system^56^. The interplay with α-syn was also investigated by crossing *Atp13a2* null mice with either mice lacking or overexpressing α-syn. In these α-syn/*Atp13a2* double knockouts, no change in the neuropathology was found compared to simple Atp13a2 null mice, while loss of Atp13a2 function exacerbates the sensorimotor phenotype in α-syn mice in an age and sex-dependent manner^73^. In 2024, an *Atp13a2* KO mouse model injected with α-syn pre-formed fibrils (PFF) of α-syn in the striatum was used to induce α-syn-related PD pathology, a strategy implemented in the present study through viral-based A53T α-syn overexpression. This work also showed that *Atp13a2* depletion does not exacerbate α-syn pathology and nigrostriatal neurodegeneration^59^. In 2024, two similar approaches based on viral vector technology were published independently^31–33^. In the study by the Moore group, an elegant recent model using recombinant AAV-Cre recombinase injection in the SN of LoxP-flanked *Atp13a2* mouse strain allowed a more precise and delineated depletion, restricted to the SN. This strategy bypasses compensatory mechanisms that can prevent neurodegeneration of the nigrostriatal pathway, as observed in other constitutive ATP13A2 KO models like ours^31^. Indeed, this adult-onset deletion of *ATP13A2* in mice induced a dopaminergic depletion up to 50% in the striatum and 35% in the SN after 10 months. This finding was replicated by a non-human primate KRS model generated by our group through the intranigral injection of a lentiviral vector expressing an ATP13A2 small hairpin RNA^32^. Both studies reinforce the idea that compensatory processes or mechanisms involved during embryonic development prevent the full expression of the deleterious consequences of ATP13A2 loss in the brain of germline deletion of *Atp13a2* in mice and rats. Here, the characterization of this new *Atp13a2* KO model in the rat fills an unmet need in the animal model field and extends the possibilities for modeling these neurodegenerative and ALP-related disorders and for studying the different processes involved in the pathology and pathogenesis caused by ATP13A2 depletion. While not perfect analogs, animal models can help overcome certain limitations of human data. To date, the literature provides no robust evidence of sex-specific differences in clinical presentation or disease progression in ATP13A2-related disorders, including KRS. Currently, the literature does not report systematic analyses of sex-specific differences in patients with *ATP13A2* mutations, likely due to the rarity of cases (fewer than 50 globally) and the predominance of single-family or small case series. No statistically meaningful sex bias in age at onset, symptom severity, or progression has been identified, with both affected males and females showing largely overlapping clinical features. Briefly, fewer than 50 KRS cases have been described globally, and case series typically include only a handful of subjects (e.g., five individuals in the original Jordanian pedigree: 4 females, 1 male)^74^. In an individual clinical series study, such as those in Ramirez et al. 2006, list both sexes but do not analyze differences in onset age, severity, or symptom patterns between males and females^75^. In one study of *ATP13A2* mutation carriers in a Chilean family, both heterozygous male and female carriers showed subtle extrapyramidal signs; no sex-specific differences in imaging or motor manifestations were reported^75^. Broader phenotype expansion studies (e.g., relating to ALS‑like or spastic paraplegia phenotypes) do not stratify findings by sex^14^. A small clinical report identified two unrelated patients with psychiatric-dominant presentation (e.g., autism spectrum, paranoid psychosis). Neither report emphasized nor compared sex-based differences in phenotype^76^. Overall, because of the rare nature of KRS (fewer than 50 cases) and the lack of demographically diverse cohorts, there’s simply insufficient statistical power to detect or claim sex-dependent effects. Further studies are needed to extend our observations, and pending questions await future investigations. Relying on recent developments in the field, the development of a rat model with conditional *Atp13a2* depletion (i.e., homozygous *Atp13a2*-floxed KO rats) induced soon after birth or in adulthood might be interesting in the study of KRS and PD, with the aim and hope of inducing dopaminergic neurodegeneration, enabling a better replication of the symptoms observed in patients. Also, further investigation of lysosomal function and characteristics in this new model could permit a better interpretation of the obtained results, such as investigating polyamine levels through metabolomic approaches, and their role in PD, KRS, and beyond. Moreover, the characterization of the impact on spinal integrity, in which lipofuscin accumulation was already observed in an *Atp13a2* KO mouse model^77^ as well as brain iron accumulation in KRS patients^11^, remains to be elucidated in this rodent model and can be a challenging point in better understanding the mechanisms induced by the ATP13A2 dysfunction in KRS. Overall, the first *Atp13a2* KO rat has interesting features for studying KRS and ALP-associated pathologies.

## Methods

### Generation of the ATP13A2 knockout rat model

Sprague Dawley rats were obtained from Charles River Laboratories (Wilmington, MA, USA). All animal care procedures were approved by the Animal Experimentation Ethics Committee of the Pays de la Loire region, France, per the French National Research Council guidelines for the Care and Use of Laboratory Animals (APAFIS #692). The CRISPR-Cas9 strategy was made in order to knock out all ATP13a2 isoforms. The first known 5’ alternative splicing occurs at the level of G463 (V154) at the end of exon 5. In consequence, a deletion of exons 4, 5, and 6 was considered by targeting the end of intron 3-4 and the beginning of intron 6-7. The single guide RNA (sgRNA) cr937 (aggatggaggctctttggaaAGG) and 940 (gagatggtgtggccaagactGGG), designed (targeted sequence in lowercase letters and PAM in uppercase letters) and produced by the facility TACGENE (Paris, France), were used to remove exons 4, 5, and 6, leading to a frameshift and a premature termination codon in exon 7. The two sgRNAs (0.2 mM for each sgRNA) and spCas9 protein (3 μM) were incubated at room temperature for 10 min to allow RNP complexes to form and then kept at 4 °C until microinjection. Process and microinjection were performed by the TRIP Platform as previously described^78^. Briefly, zygotes were collected from prepubescent (4–5-week-old) Sprague Dawley donor female rats, superovulated by injection of pregnant mare serum gonadotropin (30 IU, i.p.; Merck GmbH, Darmstadt, Germany) and, 48 h later, of human chorionic gonadotropin (20 IU; Merck), which were mated with Sprague Dawley fertile males. One-cell-stage fertilized embryos were sequentially microinjected into the male pronucleus and the cytoplasm. The same day, surviving embryos were implanted within the oviduct of pseudo-pregnant females (0.5 days postcoital) and grown until full term.

### Genotyping

Tail biopsies from 8–10-day-old rats were digested overnight at 56 °C in 500 μl of tissue digestion buffer (0.1 M Tris·HCl pH 8.3, 5 mM EDTA, 0.2% SDS, 0.2 M NaCl, 100 μg/ml proteinase K). The CRISPR’s nuclease-targeted regions were PCR amplified from diluted lysed samples (1/20) with a high-fidelity polymerase (Herculase II fusion polymerase; Agilent, Santa Clara, CA, USA). To detect gene editing, locus-specific primers were used, and mutations were analyzed by heteroduplex mobility assay-capillary electrophoresis (HMA-CE) and direct sequencing of PCR products.

This set of primers (rATP13a2-NewFor 5’-TGGGTGTAGTGTGCTGTCTAAATGG-3’/rATP13a2-Rev 5’-CCTCTTTCTGCCTGTGGAGCTG-3’) amplifies a 387 bp.

This set of primers (rATP13a2-For2 5’-CCTTCCCCACCTTGTGAGTGTG-3’/rATP13a2-Rev2 5’-GGGAGCCGAGGAGTCACATCTA-3’) amplifies a 521 bp.

This set of primers (rATP13a2-NewFor 5’-TGGGTGTAGTGTGCTGTCTAAATGG-3’/rATP13a2-Rev2 5’-GGGAGCCGAGGAGTCACATCTA-3’) amplifies a 1062 bp.

### mRNA extraction and qPCR

Tail biopsy samples were homogenized in Tri-reagent (Euromedex), and RNA was isolated using a standard chloroform/isopropanol protocol^79^. RNA was processed and analyzed following an adaptation of published methods^80^. cDNA was synthesized from 2 μg of total RNA using RevertAid Premium Reverse Transcriptase and primed with oligo-dT primers and random primers (Fermentas). qPCR was performed using a LightCycler 480 Real-Time PCR System (Roche Diagnostics). qPCR reactions were duplicated for each sample, using transcript-specific primers, cDNA (4 ng), and LightCycler 480 SYBR Green Master (Roche Diagnostics) in a final volume of 10 μl. The PCR data were exported and analyzed with a custom informatics tool (Gene Expression Analysis Software Environment) developed at the NeuroCentre Magendie. For the determination of the reference gene, the Genorm method was used^81^. Relative expression analysis was corrected for PCR efficiency and normalized against 2 reference genes. The glyceraldehyde-3-phosphate dehydrogenase (GAPDH) and the succinate dehydrogenase complex flavoprotein subunit A (SDHA) genes were used as reference genes. The relative expression level was calculated using the comparative (2–ΔΔCT) method^81^. Primer sequences against rat ATP13A2 (NM_001173432): R1356 (rAtp13a2for 5’-TACAACGGGAGCTTGCTGAA-3’ and rAtp13a2rev 5’-CAAGGCCTGCACTACCTCATG-3’), R1596 (rAtp13a2*for 5’-GAAAGAGGCAAAGCAGGTACT-3’ and rAtp13a2*rev 5’-CTGGGTCTCCATCCAGACATA-3’

### Ethics statement

All experimental protocols comply with the Council Directive of 2010 (2010/63/EU) of the European Community and the National Institute of Health Guide for the Care and Use of Laboratory Animals. The proposed research has received approval from the French Ethical Committee for Animal Research CE50 (agreement numbers: APAFIS#22334-2019100810024928, APAFIS #27720-2020092916011558, and APAFIS #17963-2018120610161720).

### AAV vector production

pAAV2-CMVie/hSyn-synA53TWPRE-pA (i.e., A53T), pAAV2-CMVie/hSyn-WPRE-pA (i.e., Stuffer), and pAAV2-CMVp-mCherry-GFP-LC3B-WPRE-pA (i.e., tfLC3) vectors were produced by polyethyleneimine (PEI) mediated triple transfection of low passage HEK-293 T/17 cells (ATCC; cat number CRL-11268). The respective AAV expression plasmids were cotransfected with the adeno helper pAd Delta F6 plasmid (Penn Vector Core, cat # PL-F-PVADF6) and AAV Rep Cap pAAV2/9 plasmid (Penn Vector Core, cat # PL-TPV008). AAV vectors were purified as previously described^51^. Cells are harvested 72 h post-transfection, resuspended in lysis buffer (150 mM NaCl, 50 mM Tris-HCl pH 8.5), and lysed by 3 freeze-thaw cycles (37 °C/−80 °C). The cell lysate is treated with 150 units/ml Benzonase (Sigma, St Louis, MO) for one hour at 37 °C, and the crude lysate is clarified by centrifugation. Vectors are purified by iodixanol step gradient centrifugation and concentrated and buffer-exchanged into Lactated Ringer’s solution (Baxter, Deerfield, IL) using Vivaspin 20 100 kDa cut-off concentrator (Sartorius Stedim, Goettingen, Germany). Titrations were performed at the transcriptome core facility (Neurocentre Magendie, INSERM U862, Bordeaux, France). The genome-containing particle (gcp) titer was determined at a concentration of 1 × 10^13^ gcp/ml by quantitative real-time PCR using the Light Cycler 480 SYBR green master mix (Roche, cat # 04887352001) with primers specific for the AAV2 ITRs (fwd 50-GGAACCCCTAGTGATG GAGTT-3’; rev 50-CGGCCTCAGTGAGCGA-30) on a Light Cycler 480 instrument. The purity assessment of vector stocks was estimated by loading 10 μl of vector stock on 10% SDS acrylamide gels, and total proteins were visualized using the Krypton Infrared Protein Stain according to the manufacturer’s instructions (Life Technologies).

### Rodent experiments and stereotactic injections

For the autophagy flux cohorts: Female WT and KO *Atp13a2* Sprague Dawley rats, 11 months old) received 2 µL of AAV9-CMVp-mCherry-GFP-LC3B (right hemisphere) and PBS (left hemisphere); for the synuclein cohorts: WT and KO ATP13A2 Sprague Dawley rats (female and male mixed, 3 months old) received 1 µL of either AAV9-CMVie/hSyn-synA53T-WPRE (AAV-A53TSyn) or AAV9-CMVie/hSyn-WPRE (AAV-Stuffer) virus (concentration: 1.0 × 10^13^ gcp/ml); for the neuromelanin cohorts: Thirty-five OFA Sprague Dawley rats (male, 2 months old) were injected unilaterally in the SN with 2 µl of either the AAV-hTyr (8.52 × 10^12^ vg/ml) or AAV-Stuffer (8.52 × 10^12^ vg/ml) by stereotactic delivery to the region immediately above the right SN (coordinates from bregma: AP, –5.4, ML, +/−2.2, DV, –7.8) at a flow rate of 0.2 µL/min, and the pipette was left in place for 5 minutes after injection to avoid leakage. Animals were perfused with 0.9% saline and 4% paraformaldehyde after 3 weeks. Brains were post-fixed for 1 day in 10 ml of 4% paraformaldehyde at 4 °C, cryoprotected in gradient 20% sucrose in PBS before being frozen by immersion in a cold isopentane bath (–50 °C) for at least 1 minute, and stored immediately at –80 °C until sectioning for histochemical analysis.

### Behavioral assessment

Assessment of neurodevelopmental milestones was performed using a modified version of the SHIRPA protocol adapted for transgenic rat models of neurodegenerative disorders^82^. All tests were performed daily from post-natal day 5 to 21. For eye opening, the following score was used: 0: no eye opened, 1:1 eye opened, 2: both eyes opened. For righting reflex, the pup is placed on its back, and the time taken to roll over onto its four paws is measured. For negative geotaxis, the pup was placed head downwards on a 45° inclined rough surface, and the time taken to turn 180° (head upward) was measured. For spontaneous locomotion, the pup was placed at the center of a 20 cm diameter circle (Whatman paper) and the time taken to move entirely off the circle was recorded. For cliff avoidance, the pup was placed on a horizontal platform 8 cm above the surface of the bench with its head positioned outside the platform. The time taken to fully move backward (no body part extending outside the platform) was measured. For the acoustic startle, the pup was placed on the bench. A tone was played using a bench timer, paced 10 cm away from the animal. A startle response (jerky movement and/or extension of limbs and/or head moving away from the tone) was given a score of 1.

The stepping test was performed on a 90cm-long adherent surface. Animals were acclimatized to the test room for 30 min. At test time, the animal was placed on the surface for a few seconds. The animal was handled as follows: the left front paw was released from the rest of the body and placed on the surface. The rat was then moved from the left to the right of the surface (forward movement) and then from the right to the left (backward movement). The number of steps taken for each round trip was counted. The same procedure is then applied to the right paw. The test was carried out over 4 days, with one session in the morning and one in the afternoon, at least 4 hours apart.

The single-pellet reaching task is a sensitive test of manual dexterity and a potent rehabilitative task of rat forelimbs that allows both quantitative and qualitative assessment of fine motor skills. The rats were food-restricted before each session to enhance motivation in the test. The animals were weighed and placed on a restricted feeding schedule (10–15 g standard chow/daily) 48 hours before behavioral training, reducing their body weight by 5% to 10% of their initial weight. Handle rats for several minutes per day for at least 5 days. After handling, place 4–5 sugar pellets per rat in each home cage to introduce the novel food. All animals were trained on single-pellet reaching tasks and randomly assigned to groups. Animals were placed in boxes where they could reach food pellets through a 9 mm vertical gap. The food pellet was replaced after the rat either grasped it or knocked it off the shelf. Each rat was given a 20-minute task each day.

The Locomotor Activity was assessed with the apparatus (35.5 × 23.5 cm, Imetronic), which used an array of infrared beams to determine the activity and mobility of a subject. The activity cage was incompletely divided into two compartments (front and back). Three parameters were recorded: activity, rearing, and movement between the back and front compartments. “Activity” was the total number of infrared beam breaks.

### Electrophysiological recordings

Isoflurane anesthesia was used during stereotaxic surgery to conduct electrophysiological experiments on 3, 6, and 12-month-old KO and WT rats. A glass micropipette (tip diameter = 2–3 µm, 4–6 Mohm) filled with a 2% pontamine sky blue solution in 0.5 M sodium acetate was lowered into the SN at the following coordinates: −5.4 mm from bregma; 2.1 mm from the midline. Dopaminergic neurons were identified thanks to their half action potential width ≥1.1 ms, their firing rate under 10 Hz, and their bursting patterns defined as the occurrence of two spikes with an interspike interval <80 ms^83^. The extracellular potential was recorded through these electrodes with an Axoclamp2B amplifier in the bridge mode. The extracellular potential amplified 10 times by the Axoclamp2B amplifier was further amplified 100 times and filtered (low-pass filter at 300 Hz and high-pass filter at 0.5 kHz) via a differential AC amplifier (model 1700; A-M Systems, Carlsborg, WA). Single neuron spikes were discriminated and digital pulses were collected online using a laboratory interface and software (CED 1401, SPIKE 2, Cambridge Electronic Design). Four parameters of SN dopamine neuron impulse activity were computed over 200 s epochs after a 5 min stable baseline period: 1) half spike width, 2) the basal firing rate, and 3) mean spike per burst, and 4) mean burst length. The onset of a burst was defined as the occurrence of two spikes with an interspike interval <80 ms^83^. We evaluated the amount of bursting activity by calculating a bursting coefficient (burst event frequency x number of spikes within each burst) and a coefficient of variation (defined as the ratio of the standard deviation to the mean of spike intervals). Both the frequency and the bursting rate were used to classify dopamine neurons in four subpopulations according to the previously described criteria^84^: low frequency low bursting (LFLB), low frequency high bursting (LFHB), high frequency low bursting (HFLB), and high frequency high bursting (HFHB). LFLB is characterized by a firing rate lower than 5 Hz and a % of spikes in a burst lower than 20%. LFHB is characterized by a firing rate lower than 5 Hz and a % of spikes in bursts in the range of 20-60%. HFLB has high frequency (>5 Hz) and low bursting, with a % of spikes in a burst <40%. Lastly, HFHB corresponds to high frequency (>5 Hz) and a % of spikes in a burst >40%.

### Histological analysis

To assess the integrity of the nigrostriatal pathway, TH immunohistochemistry was performed on SN and striatal sections. Briefly, sections from 3 representative levels of the striatum (anterior, medial, and posterior) and serial sections (1 of 6) corresponding to the whole SN were incubated with a rabbit monoclonal antibody raised against TH (Abcam, EP1532Y, ab137869, 1:5000) for 1 night at room temperature and revealed by an anti-rabbit peroxidase EnVisionTM system (DAKO, K400311) followed by DAB visualization. Free-floating SN sections were mounted on gelatinized slides, counterstained with 0.1% cresyl violet solution, dehydrated, and cover-slipped, while striatal sections were mounted on gelatinized slides and cover-slipped. The extent of the lesion in the striatum was quantified by OD. Sections were scanned in an Epson Expression 10000XL high-resolution scanner, and images were used in ImageJ open-source software to compare the gray level in the striatum. TH-positive SN cells were counted by stereology, blind to the experimental condition, using a Leica DM6000B motorized microscope coupled with the Mercator software (Explora Nova). The SN was delineated for each slide, and probes for stereological counting were applied to the map obtained. Each TH-positive cell with its nucleus included in the probe was counted. The optical fractionator method was finally used to estimate the total number of TH-positive cells in the SN of each rat hemisphere.

α-Synuclein pathology was assessed with a rabbit monoclonal antibody raised against α-synuclein (Cell Signaling, D37A6, 1:5000), against human α-synuclein (Abcam, MJFR1, ab138501, 1:5000) and against phosphorylated α-synuclein (Abcam, EP1536Y, ab51253, 1:5000). Briefly, 3 representative levels (anterior, medial, and posterior) of SN and striatum were explicitly identified and incubated in the same well to allow direct comparison of immunostaining intensity. Sections were incubated overnight at room temperature with the aforementioned antibodies. For pretreatment with proteinase K (PK), sections were incubated first with PK at 5 μg/ml in PBS for 20 min at room temperature before long sequential washes in distilled water and then in PBS. The signals were revealed using the anti-species peroxidase EnVision system (DAKO) followed by DAB incubation. Sections were mounted on slides, dehydrated, and cover-slipped. For human α-synuclein analysis, sections were scanned in an Epson Expression 10000XL high-resolution scanner, and images were used in ImageJ open-source software to compare the gray level of human α-synuclein in the SN and striatum. For pS129-α-synuclein analysis, sections were scanned in a high-resolution scanner (PanScan, 3D Histech) at ×20 magnification, and the quantification of pS129-α-synuclein-positive surface was estimated using a threshold detection macro on the Visiopharm software.

Inflammatory processes in the SN and the striatum were measured through GFAP/S-100 (Merck, MAB360, 1: 1,2000/ Abcam, ab7852, 1: 1000) and Iba1 (Abcam, ab5076, 1: 1000). Striatal sections of all animals were incubated overnight with a mix of mouse antibodies raised against GFAP and S-100 for the astroglial staining and a rabbit anti-Iba1 antibody for the microglial staining. These signals were revealed with an anti-species peroxidase EnVision system (DAKO) followed by DAB incubation. Sections were mounted on slides, dehydrated, and cover-slipped. Sections were scanned in a high-resolution scanner (PanScan, 3D Histech) at ×20 magnification, and an immunostaining-positive surface quantification estimated the quantification of GFAP-positive astrocytic staining or Iba1-positive microglial staining at regional levels with the Visiopharm software.

For imaging of lipofuscin, untreated free-floating nigral slices were mounted with a Vectashield Antifade Mounting Medium to observe lipofuscin autofluorescence. Z-stack images were acquired using a Leica DM6 CFS TCS SP8 confocal microscope at ×63 magnification, with an excitation of 500 nm and an emission of 600-650 nm. Analysis was performed through Fiji/ImageJ. Staining of NM granules in 50-µm-thick rat brain sections was performed using the Masson-Fontana Staining Kit (DiaPath), as previously described in 46. Brightness and contrast rules were applied to the RGB pictures to optimize details without any image saturation. The color thresholding tool was then used to select the threshold corresponding to the NM staining.

For the ultrastructural examination by electron microscopy, rats were perfused transcardially with 4% paraformaldehyde (PFA) and 2.5% glutaraldehyde in 0.1 M phosphate buffer (PB) pH 7.4. Brains were removed, post-fixed for 12 h at 4 °C in 4% PFA and cut into 60-μm-frontal section with vibratome (Leica, VT1000S Germany). The sections were post-fixed in 1% osmium tetroxide in 0.1 M PB (pH 7.4), dehydrated in an ascending series of ethanol dilutions that also included 70% ethanol containing 1% uranyl acetate. They were treated with propylene oxide, impregnated in resin overnight (Durcuan ACM Sigma-Aldrich), mounted on glass slides, and cured at 60 °C for 48 hours. Areas of interest (substantia nigra pars compacta were cut from the sections and glued to blank cylinders of resin. Ultra-thin sections made with an ultramicrotome Ultracut (S Leica) were collected on piolofororm-coated single-slop copper grids (Electron Microscopy Sciences, FCF2010-Cu), stained with lead acetate and examined with a HITACHI-H7650 (HITACHI Ltd., Tokyo, Japan) electron microscope. Quantitative analysis was performed according to the previously described procedure^12,85^. Ultrastructural and morphometric measurements were conducted in digital images using Fiji software. Images were acquired with a magnification of ×12,000 (0.0042 µm-pixel size) and processed identically. All analyses were conducted by an individual blinded to all cell types. Autophagic vacuoles were identified using previous criteria^86^, and were then classified and counted on electron micrographs from different genotypes in each experimental group. Quantitative analysis was carried out on the whole cytosol area, and results were expressed as the number of vacuoles per square micrometer.

### Primary culture of mouse dopaminergic neurons and transient transfection

Rat dopaminergic neurons were cultured as described in ref. ^87^. Briefly, pregnant female mice (C57BL/6JRj, Janvier Labs) of 14 days gestation were killed by CO2 inhalation, and the fetuses were removed from the uterus. The embryonic midbrains were removed and placed in an ice-cold medium of Leibovitz 15 (L15; Dutscher, ref: P04-27055) containing 1% of Penicillin-Streptomycin (PS; Thermofisher, ref: 15140122) and 1% of bovine serum albumin (BSA; Dutscher, ref: P06-1391100). Only the ventral portions of the mesencephalic flexure were used for the cell preparation, as this region of the developing brain is enriched in dopaminergic neurons. The midbrains were dissociated by trypsinization for 20 minutes (min) at 37 °C (Trypsin EDTA 1×; Fisher Scientific, ref: 25300-54). The reaction was stopped by the addition of Dulbecco’s modified Eagle’s medium (DMEM; Dutscher, ref: P10-023100) containing DNase I grade II (0.1 mg/ml; Dutscher, ref: P60-37780100) and 10% of fetal calf serum (FCS; Fisher Scientific, ref: 10270106). Cells were then mechanically dissociated by 3 passages through a 10 mL pipette. Cells were then centrifuged at 180 × *g* for 10 min at +4 °C on a layer of BSA (3.5%) in L15 medium. The supernatant was discarded, and the cell pellets were re-suspended in a defined culture medium consisting of Neurobasal Plus (Fisher Scientific, ref: A3653401) supplemented with 2% of B27 Plus (Fisher Scientific, ref: A3653401), L-glutamine (2 mM; Dutscher, Ref: P04-80100), 1% of PS, 10 ng/mL of BDNF (Fisher Scientific, ref: 450-02) and 1 ng/mL of Glial Derived Neurotrophic factor (GDNF; Fisher Scientific, ref: 450-10). Viable cells were then counted in a Neubauer chamber using the trypan blue exclusion test. The cells were seeded at a density of 35,000 cells/well in a black 96-well plate with transparent bottom and Poly-D-Lysine-coated surfaces and cultured at 37 °C in a humidified air (95%)/CO2 (5%) atmosphere. Half of the medium was changed every 2 days with fresh medium. After 5 days of culture, astrocytes are present in the culture and release growth factor, allowing neuron differentiation. In this condition, 2–5% of neurons are dopaminergic neurons. After 7 days of culture, cells were grown to 70% confluence and transfected using Lipofectamine 3000 (Thermo Fischer, ref: L3000001). Transfection was performed according to the manufacturer’s protocol. Briefly, for each well of a 96-well plate, the lipofectamine mix was prepared by diluting 0.2 μl of Lipofectamine® 3000 reagent in 5 μl of Opti-MEM® Medium. The plasmid mix was prepared by diluting 0.1 or 0.2 μg of plasmid and 0.2 μl of P3000™ Reagent in 5 μl of Opti-MEM® Medium. The lipofectamine mix and plasmid mix were prepared separately, mixed together, and incubated for 10 min at room temperature before adding to the cells. After 6 h of transfection, the medium was changed, and cells were grown for 24 h before treatment with 500 nM synuclein PFF for 4 days. 6 wells of a 96-well plate were used for each condition.

Monomeric α-Syn (PROTEOS RP010) solutions at 5 mg/mL in 10 mM Tris-HCl, NaCl 50 mM, pH 7.60) were seeded with 5% of fibrils. The samples were loaded in a ThermoMixer (Eppendorf, Hamburg, Germany) in a 24-position 1.5 mL Eppendorf tube holder equipped with a heating lid. The temperature was set to 37 °C, and continuous shaking at 2000 rpm proceeded for 7 days.

After 4 days of intoxication with PFF synuclein, primary cell culture was fixed by a solution of 4% paraformaldehyde (Alpha Aesar, ref J19943) for 20 min at room temperature, permeabilized and non-specific sites were blocked with a solution of phosphate-buffered saline (PBS; VWR; ref: L0615-500) containing 0.1% of saponin (Sigma; ref: S7900) and 1% FCS for 15 min at room temperature. Cells were incubated with a chicken tyrosine hydroxylase polyclonal antibody (Thermo Fischer, ref: PA5-143583,1/1000), a mouse phosphorylated alpha-synuclein pS129 antibody (Abcam, ref: ab184674, 1/500), and a rabbit anti-LC3B polyclonal antibody (Cell Signaling, ref: 2775S, 1/100), in a solution of PBS with 0.1% of saponin and 1% FCS overnight at 4 °C. Staining was revealed with the addition of an Alexa Fluor 488 goat anti-chicken IgG (Thermo Fischer, ref: A11039, 1/400), an Alexa Fluor 647 goat anti-mouse IgG (Thermo Fischer, ref: A21235, 1/400), an Alexa Fluor 568 goat anti-rabbit IgG (Thermo Fischer, ref: A11011, 1/400) and a fluorescent dye used to stain nuclei (Hoechst, Sigma; ref: B2261) in PBS with 0.1% saponin and 1% FCS for 1 hour at room temperature.

For each analysis based on immunofluorescence, images were taken automatically with an automated microscope InCell2200. The same corresponding area was acquired in each well, avoiding any experimenter impact on the acquisition process. Analyses were then done automatically using the same program for all treatment conditions, so the same selected parameters were applied with the Developer software to avoid the impact of the experimenter on the analysis process. The sum of each measured parameter was calculated using 20 images per well. The value obtained for each condition was then normalized to control conditions. Analysis of the number of dopaminergic neurons (TH-positive neurons), the neurites length of dopaminergic neurons (TH-positive neurites), the phosphorylated α-synuclein area in dopaminergic neurons (pS129-α-synuclein and TH colocalization area), and the colocalization of phosphorylated α-synuclein with LC3B foci in mesencephalic cells (pS129-α-synuclein and LC3B colocalization area) was performed using the Image Analysis Software (Toolbox processing Developer v1-9-2, GE Healthcare). To measure the area of phosphorylated α-synuclein in TH-positive cells, as well as pS129-α-synuclein and LC3B colocalization area in mesencephalic cells, algorithmic segmentation approaches to mask cell bodies were performed and followed by the identification of pS129-α-synuclein or LC3B within the mask. Data were expressed as the mean ± standard error of the mean (*n* = 6 per condition) and analyzed using one-way ANOVA followed by Dunnett correction.

For immunofluorescence and Image Analysis, sections were permeabilized for 1 hour in a 4% donkey serum/PBS blocking buffer containing 0,2% Saponin and incubated overnight at 4 °C with the following primary antibodies diluted in a 1% Donkey serum/PBS buffer: mouse anti-TH (Merck, MAB318, 1:2000), rat anti-LAMP2 (Abcam, ab193, 1:1000) and rabbit anti-human α-synuclein (Abcam, MJFR1, ab138501, 1:5000), rabbit anti-S129 phosphorylated α-synuclein (Abcam, EP1536Y, ab51253, 1:5000), rabbit anti-spermine (Abcam, ab26975, 1:2000), rabbit anti-spemidine (Abcam, ab7318, 1:2000). Following incubation with primary antibodies, tissues were washed with PBS 3 times for 10 minutes each and incubated for 1.5 hours at room temperature with a combination of corresponding donkey anti-species IgG conjugated to AlexaFluor probe (Invitrogen, 1:400). Tissues were then washed with PBS and incubated with Hoechst (10 µM) for 5 minutes at room temperature before being mounted in mounting media (Vectashield). Illustrative images were acquired using a wide-field Olympus Epifluorescent Microscope (BX3-CBH) coupled with a Hamamatsu camera (ORCA-Flash 4.0 LT). All image acquisitions and analyses were performed blinded to the researcher. Images were deconvolved using the cellSens Dimension software. For LAMP2 count in TH-positive cells, image analysis was performed in Fiji/ImageJ. Cells were segmented by manual cell selection using the TH channel. The mask was then applied to the hSyn channel to confirm the hSyn overexpression in the selected TH-neuron for the A53T-Syn group. The mask was finally applied to the LAMP2 channel, where LAMP2 staining was quantified by automatic thresholding followed by binarization. Total staining was then normalized per cell. Colocalizations were analyzed through semi-automatic quantification with an ImageJ macro for fluorescent markers present on a focal plan in confocal fluorescent images called Bioloc3D 10.5281/zenodo.8087692.

### Biochemical analysis

Western blots were performed from 20 μg of protein separated by SDS-polyacrylamide gel electrophoresis and transferred to 0.2 μm nitrocellulose membrane (Bio-Rad). Incubation of the primary antibodies was performed overnight at 4 °C with ATP6V1A Polyclonal antibody (cat. number: 17115-1-AP, Proteintech, 1:1000), anti-LAMP2 (L0668, Sigma-Aldrich, 1:1000). Anti-actin (Sigma-Aldrich, 1:5000) or anti-tubulin (T5168, Sigma-Aldrich, 1:5000) was used to control equal loading. Appropriate secondary antibodies coupled to peroxidase were revealed using an Immobilon Western Chemiluminescent HRP Substrate kit (Immobilon Western, Chemiluminescent HRP Substrate, Millipore). Chemiluminescence images were acquired using the ChemiDoc XRS+ system (Bio-Rad). Signals per lane were quantified using ImageJ, and a ratio of signal on loading per animal was calculated and used in statistical analyses.

For the isolation of lysosomal fractions and CatD activity assay, fresh ventral midbrain samples from WT- or KO rats at 16 months old were dissected. The tissue was homogenized immediately in buffered sucrose (Sigma-Aldrich, S9378; 4 mM HEPES, 150 mM sucrose, pH 7.4) and then subjected to differential centrifugation. All subsequent steps of the lysosomal isolation were performed according to the manufacturer’s description (Lysosome Isolation Kit, LYSISO1; Sigma). For the LAMP2 immunoblot, equal loading was verified by Ponceau red staining of the membrane. CatD activity was measured in the lysosomal-enriched fraction using a fluorometric CatD activity assay kit (Abcam, ab65302) under the manufacturer’s instructions. Fluorescence was measured on a FLUOstar Optima microplate analyzer (BMG Labtech, Champigny-sur-Marne, France).

### SR-XRF microscopy elemental mapping of brain tissue cryosections

The synchrotron experiments were carried out at the Diamond Light Source, Harwell Science and Innovation Campus (Didcot, UK) with 3-GeV energy of the storage ring and 300-mA currents with top-up injection mode. All SR-XRF microscopy investigations reported herein were carried out on the microfocus spectroscopy beamline (I18). The micro-XRF elemental mapping was acquired at room temperature with an incident x-ray energy set to 12 keV using a Si(111) monochromator. This resulted in an x-ray photon flux of 2 × 10^11^ photons/s. The SN cryostat section (50 μm in thickness) of each animal was collected from free-floating sections and mounted onto an x-ray transparent metal-free 4-μm-thick Ultralene foil (SPEX CertiPrep, Metuchen, NJ, USA) secured to a customized polyetheretherketone (PEEK) holder, ensuring contamination-free samples and reduced x-ray scattering contribution. The samples were then left and kept at room temperature in a moisture-free box until used for analysis. The samples were affixed to a magnetic plate connected to the sample stage of the microfocus beamline. The four-element Si drift Vortex ME4 energy dispersive detector (Hitachi High-Technologies Science America), with Xspress 3 processing electronics, was operated in the 90° geometry; hence, it minimizes the background signal. The sample-detector distance was fixed (75 mm). The sample was held at 45° to the incident X-ray beam and rastered in front of the beam while the XRF spectra were collected. An area of 500 μm by 500 μm within the SN was mapped for each sample with a step size that matches the beam size (5 μm) and a dwell time of 1 s per pixel due to the low concentration of the element. A thin (100 μm) pellet of the National Institute of Standards and Technology (NIST) standard reference materials SRM1577c (bovine liver material, NIST, Gaithersburg, MD, USA) was measured to calibrate experimental parameters, as well as a thin-film XRF reference material (AXO DRESDEN GmbH) with nanoscale uniform mass depositions in the range of ng/mm^2^. This was followed by elemental quantification calculated using the Fundamental Parameters (FP) approach through the open-source software PyMCA^88^, in which the reference material and the sample are modeled in terms of main composition, density, and thickness. The fluorescence spectrum obtained from each pixel was fitted, the elemental concentration (micrograms per gram of dry weight or parts per million) maps were generated, and an average elemental concentration of the SN regions was obtained.

### Neurotransmitter analysis

Brain patches were dissected on an ice-cold plate, weighed, put into 1.5-ml Eppendorf tubes, and stored at −80 °C until HPLC analysis. Samples were homogenized in methanol/water (85:15%, v/v) for GABA and glutamate quantification and in 0.1 M perchloric acid solution for dopamine, DOPAC, HVA, and 3-MT quantification. The homogenized samples were centrifuged at 12,000 rpm for 10 min at 4 °C, and the supernatant was collected. Glutamate and GABA content in the samples was measured by high-performance liquid chromatography (HPLC) coupled with fluorometric detection (FP-2020 Plus fluorimeter, Jasco, Tokyo, Japan) after precolumn derivatization with o-phthaldialdehyde/mercaptoethanol (OPA) reagent^89,90^. Thirty microliters of OPA reagent were automatically added to 28 μl of the sample by a refrigerated (4 °C) autosampler (Triathlon, Spark Holland, Emmen, The Netherlands). Fifty microliters of the mixture were injected onto a 5-C18 Hypersil ODS column (3 × 100 mm; Thermo Fisher Scientific, USA) perfused at 0.48 ml/min (Jasco PU-2089 Plus Quaternary Pump; Jasco, Tokyo, Japan) with a mobile phase containing 0.1 M sodium acetate, 10% methanol, and 2.2% tetrahydrofuran (pH 6.5). Chromatograms were acquired and analyzed using ChromNAV software (Jasco, Tokyo, Japan). Dopamine and dopamine metabolites content in the samples was measured by HPLC coupled to electrochemical amperometric detection (DECADE II, Antec Scientific, Alphen a/d Rijn, The Netherlands). Twenty microliter samples were automatically injected by a refrigerated (4 °C) autosampler (Triathlon, Spark Holland, Emmen, The Netherlands) onto a C18 Synergi column (75 × 2 mm, 4 μm, Phenomenex, Torrance, CA, USA) perfused with a mobile phase composed of sodium phosphate 0.150 M, 0.1 mM EDTA, octanesulfonic acid 0.6 M and methanol 17%. Chromatograms were acquired and analyzed using Clarity software (Antec Scientific, Alphen a/d Rijn, The Netherlands).

### Statistical analysis

Statistical analyses were performed with Prism 10 (GraphPad Software Inc.). Comparisons among means were performed using unpaired Student’s *t* test or two-way ANOVA, followed, if appropriate, by pairwise comparison between means by Tukey post hoc test. All values are expressed as the mean ± SEM unless specified otherwise in the figure legend. Each dot in the scatter plot represents one individual. Statistical significance was set at *p* < 0.05.

## Supplementary information

Supplementary information

Supplementary information

## Data Availability

All data generated or analysed during this studyare included in this published article and can be found in supplementary dataset 1.
