# Supplementary material for: Phenotypic characterization of an Atp13a2 knockout rat model of Parkinson’s disease
Source: NPJ Parkinsons Dis. 2025 Nov 18;11:321. doi: 10.1038/s41531-025-01171-0 (PMC12627524; doi:10.1038/s41531-025-01171-0)
Supplement: Supplementary file 1 — Supplementary information [file 41531_2025_1171_MOESM1_ESM.pdf]

Supplementary Figures

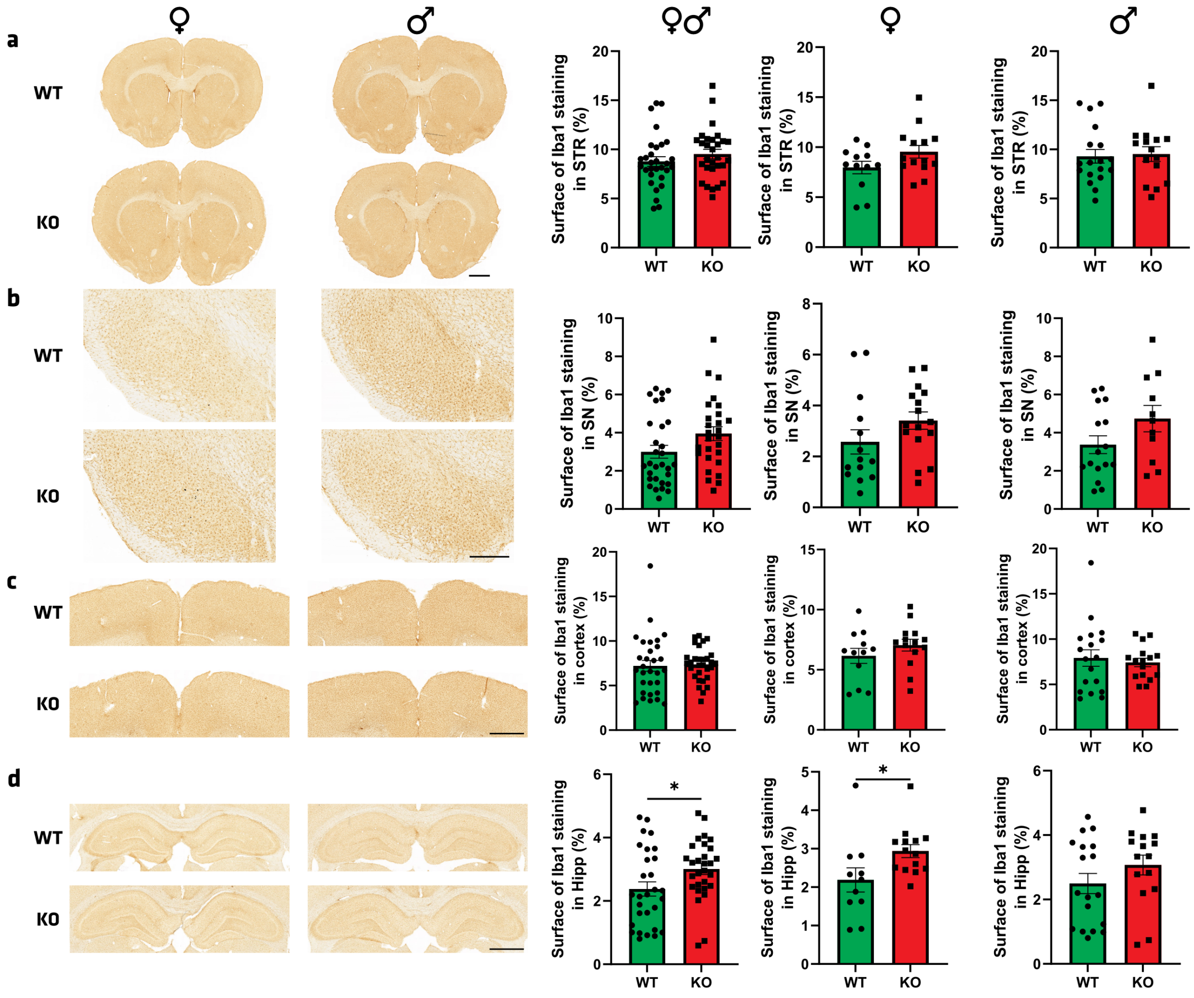

Supp. Figure 1. Loss of *Atp13a2* does not elicit microglial-mediated neuroinflammation. The surface of microglia through Iba1 staining in the (**a**) striatum (STR), (**b**) the substantia nigra (SN), (**c**) the cortex, and (**d**) the hippocampus (Hipp). Data are expressed as mean +/- SEM, n=12-19 per group. *$p < 0.05$, unpaired Student's t-test. Scale bar A, C, D = 2 mm; B = 500 µm.

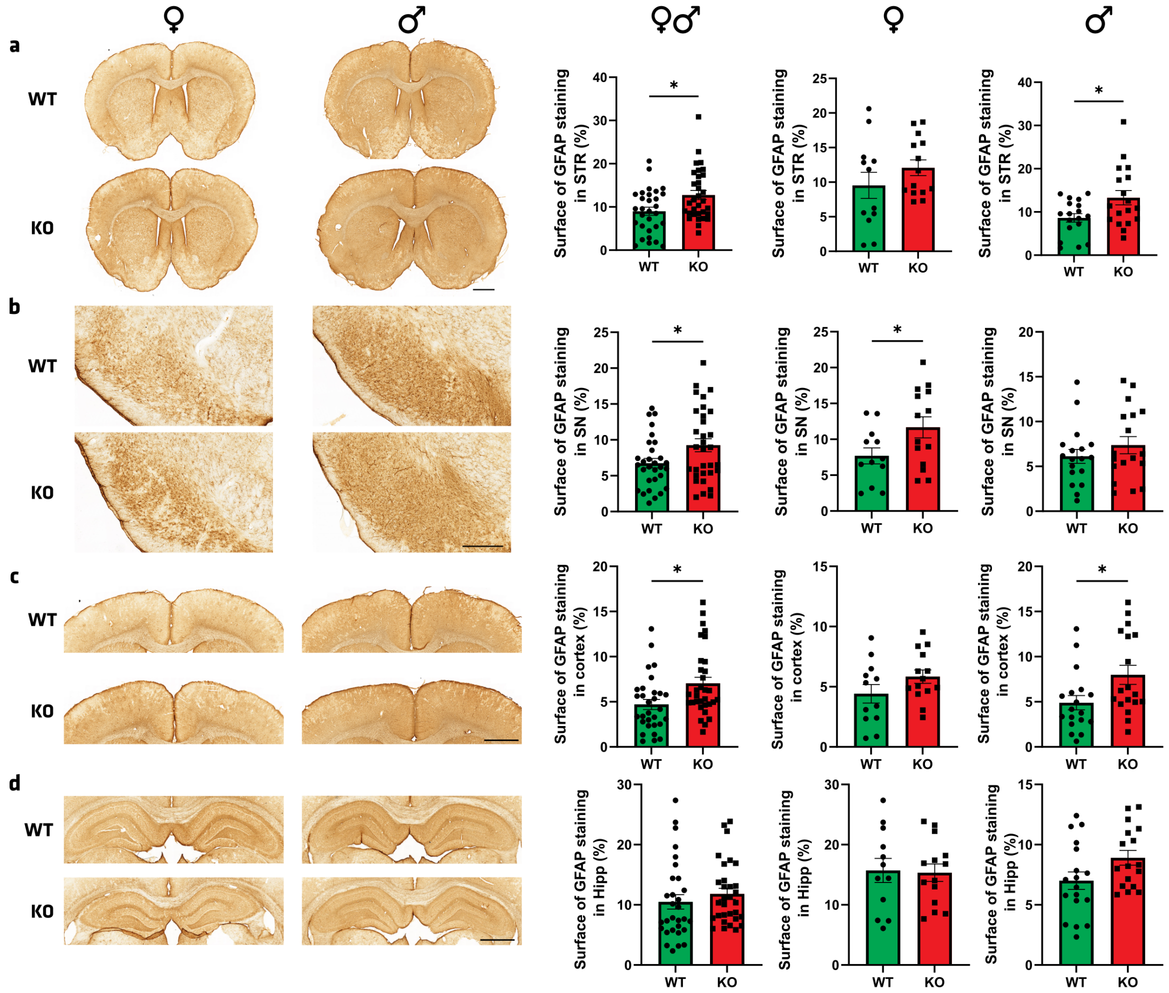

Supp. Figure 2. Astrogliosis assessment in *Atp13a2* KO rats. The surface of astrocytes revealed by GFAP/S100 staining in the (**a**) striatum (STR), (**b**) the substantia nigra (SN), (**c**) the cortex, and (**d**) the hippocampus (Hipp). Data are expressed as mean +/- SEM, n=12-19 per group. *$p < 0.05$, unpaired Student's t-test. Scale bar A, C, D = 2 mm; B = 500 µm.

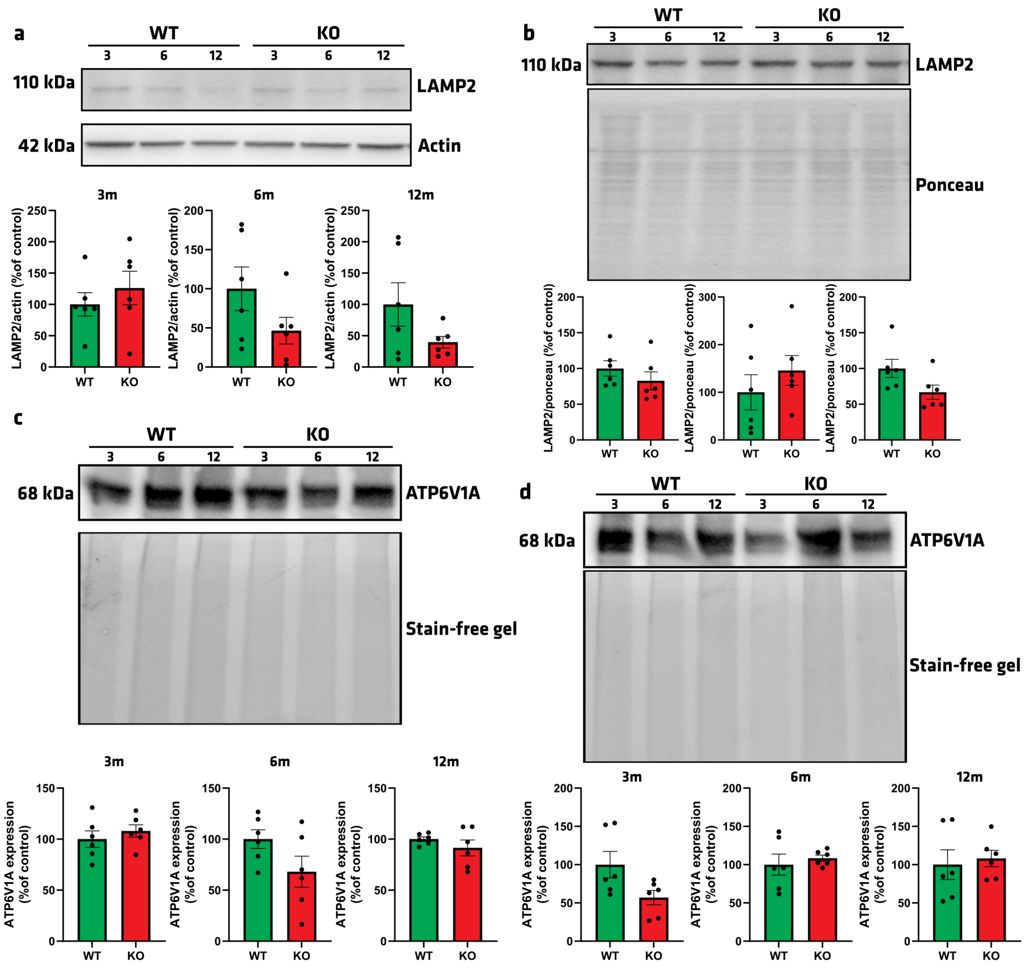

Supp. Figure 3. Analysis of ALP-related proteins in the SN, STR, and SN-lysosomal fraction of 16-month-old rats. (**a**) Measurement of the lysosomal marker lysosomal-associated membrane protein 2 (LAMP2) over actin in the SN. (**b**) LAMP2 over total protein revealed by ponceau counterstaining. (**c-d**) The total levels of ATP6V1A in striatal (**c**) and nigral (**d**) lysates were unaffected between WT and KO rats. Data are expressed as mean +/- SEM.*p < 0.05, unpaired Student's t-test.

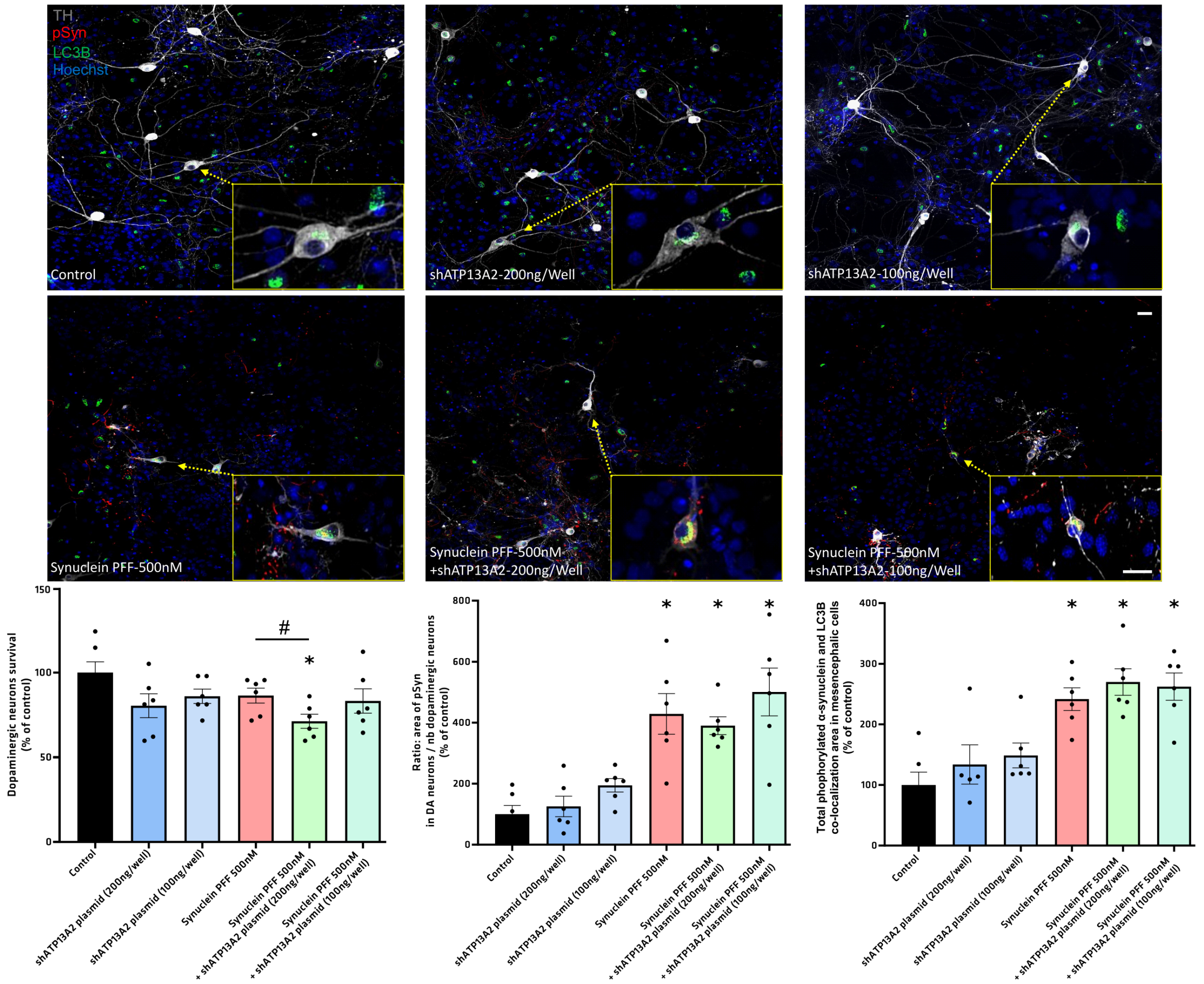

Supp. Figure 4. Modulation of the ATP13A2 expression in primary culture of mouse dopaminergic neurons through transient transfection. Illustrative images and quantification of the dopaminergic neuron survival, the ratio of the pSyn-staining area over the number of dopaminergic neurons (grey-TH), and the colocalization of pSyn (red) and LC3B (green) in mesencephalic cells, depending on the treatment applied with shATP13A2 plasmid (at 100 or 200ng/well) and synuclein PFF 500 nM. Data are expressed as mean +/- SEM.*$p < 0.05$ vs. control. #$p < 0.05$. Tukey's post hoc test followed a significant two-way ANOVA. Scale bar = 20 µm.

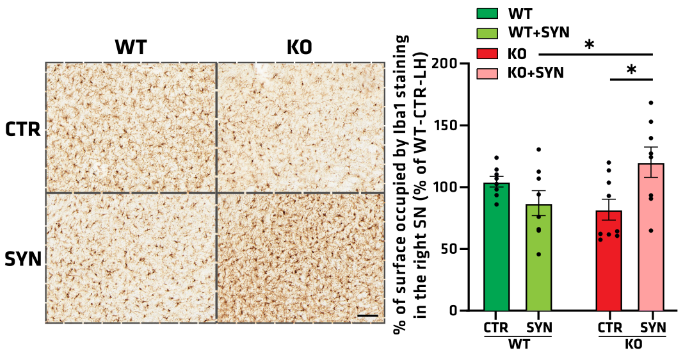

Supp. Figure 5. Microglial reactivity in the KO+SYN group. The surface of microglia through Iba1 staining in the SN of *AAV-A53T / AAV-Stuffer* cohort. Data are expressed as mean +/- SEM, n=12-19 per group. *$p < 0.05$, Tukey's post-hoc test following a significant two-way ANOVA. Scale bar = 100 µm.

Supplementary Dataset 1. Raw data supporting the findings of this study.
